# Intravenously delivered multilineage-differentiating stress-enduring cells dampen hyperoxia-induced lung injury in a rat model with features of bronchopulmonary dysplasia

**DOI:** 10.1186/s13287-026-05086-2

**Published:** 2026-09-04

**Authors:** Ryosuke Miura, Atsuto Onoda, Azusa Okamoto, Toshihiko Suzuki, Takahiro Kanzawa, Sakiko Suzuki, Kazuto Ueda, Shinobu Shimizu, Yoshiyuki Takahashi, Masahiro Hayakawa, Yoshiaki Sato

**Affiliations:** 1 Division of Neonatology, Center for Maternal-Neonatal Care, Nagoya University Hospital, 65 Tsurumai-cho, Showa-ku, Nagoya, Aichi 466-8560 Japan; 2 Faculty of Pharmaceutical Sciences, Sanyo-Onoda City University, 1-1-1 Daigakudori, Sanyo-Onoda, Yamaguchi 756-0884 Japan; 3 Department of Advanced Medicine, Nagoya University Hospital, 65 Tsurumai-cho, Showa-ku, Nagoya, Aichi 466-8560 Japan; 4 Department of Pediatrics, Nagoya University Graduate School of Medicine, 65 Tsurumai-cho, Showa-ku, Nagoya, Aichi 466-8560 Japan

**Keywords:** Muse cells, BPD, Mesenchymal stromal cell, Hyperoxia-induced neonatal lung injury model

## Abstract

**Background:**

Neonatal bronchopulmonary dysplasia (BPD) is a lung injury caused by various factors, including intrauterine inflammation, mechanical ventilation, and oxidative stress. BPD results in serious respiratory and neurological dysfunctions and mortality. Recently, some clinical trials have commenced using intravenous delivery of donor-derived multilineage-differentiating stress enduring (Muse) cells. In the present study, we aimed to investigate the therapeutic effects of human Muse cells in hyperoxia-induced neonatal lung injury in a rat model with features of bronchopulmonary dysplasia.

**Methods:**

Rats were put into the incubator within 24 h from birth to expose to hyperoxia (83%) until postnatal day 15. Muse and non-Muse cells, obtained from the bone marrow-mesenchymal stromal cells (MSCs) as stage-specific embryonic antigen-3 (SSEA-3)+ and −, respectively, were administered slowly via the right external jugular vein or trachea (Muse cells only) on postnatal day 5. For the vehicle groups, only the acetic acid Ringer's solution was administered.

**Results:**

Respiratory function, histological findings, and inflammatory parameters did not differ significantly between intravenous and intratracheal administration. In contrast, body weight gain and survival were worse following intratracheal administration. Intravenous administration of Muse cells resulted in superior amelioration of lung tissue injury, inflammation, and pulmonary hypertension compared with non-Muse cells. We also confirmed the engraftment of Muse cells in the lung tissues. Proteomic profiling identified hyperoxia-induced lung injury associated changes in the abundance of proteins annotated to cell adhesion and coagulation/fibrinolysis-related pathways, and Muse cell administration was associated with differential abundance of subsets of these proteins.

**Conclusions:**

Our findings suggest that intravenously transplanted Muse cells provide functional benefits in hyperoxia-induced lung injury in a rat model with features of bronchopulmonary dysplasia.

**Supplementary Information:**

The online version contains supplementary material available at https://doi.org/10.1186/s13287-026-05086-2.

## Background

Neonatal bronchopulmonary dysplasia (BPD) is a lung injury caused by various factors, including intrauterine inflammation, mechanical ventilation, and oxidative stress [1]. With the advancement in neonatal care, the survival rate of infants born with a birth weight of < 1500 g [very low birth weight (VLBW) infants] has increased to > 90% [2]. However, neonatal BPD remains a serious complication affecting in approximately 40% of surviving infants born at ≤ 28 weeks’ gestation, and its incidence has been relatively stable over the last few decades [3]. Many infants with severe BPD die due to respiratory distress, and even those who are discharged alive require home ventilators and/or home oxygen therapy. Even after hospital discharge, BPD infants have a high likelihood of hospitalization for respiratory infections, and their medical costs are high [4]. Additionally, BPD can lead to impaired respiratory function persisting into adolescence [3] and a low intelligence quotient score in school-aged children [5]. Currently, no definitive treatment exists beyond ventilator management. Systemic administration of steroids may have some short-term treatment effects, yet there are concerns about their long-term neurological outcome [6, 7], even with low-dose administration [8]. Therefore, the development of new therapies for BPD is an urgent issue.

Recently, stem cell therapy has brought “hope” to patients with various diseases for which no adequate treatment has been available. Even in perinatal and neonatal medicine, numerous animal studies across various diseases have been published [9–14]. As for BPD, mesenchymal stromal cells (MSCs) are the primary cell source for stem cell therapy [15]. A previous meta-analysis of preclinical studies have revealed that MSCs considerably improved alveolarization and ameliorated pulmonary hypertension, lung tissue inflammation, fibrosis, angiogenesis, and apoptosis [16]. Additionally, more than 10 clinical trials have been started worldwide [17], and the results for some of these have been already reported [18–21]. However, the effect of MSC therapy in these clinical trials did not demonstrate sufficient efficacy in terms of reducing the risk for mortality or moderate/severe BPD [21]. Therefore, to enhance the treatment effects, therapy modifications and/or other strategies should be considered.

Multilineage-differentiating stress enduring (Muse) cells, which make up a small fraction of the total MSCs [22], display endogenous pluripotent-like property and express pluripotent surface marker stage-specific embryonic antigen-3 (SSEA-3) [23]. Muse cells express sphingosine-1-phosphate (S1P) receptor 2 and selectively home into the damaged sites by sensing S1P, induced by the injured cells [24, 25]. Importantly, Muse cells also possess phagocytic activity that allows them to recycle transcription factors from the damaged cells and rapidly differentiate into the same cell type at the homed tissues, thereby replenishing injured cells and repairing the tissues [9, 24–29]. Therefore, Muse cells do not require artificial induction to a pluripotent state or artificial differentiation induction into the target cell type. Besides replacing the damaged cells, the therapy using Muse cells has paracrine effects, as it produces various trophic factors [9]. Moreover, since Muse cells have an immunosuppressive property similar to that of the placenta, specifically the expressions of human leukocyte antigen (HLA)-G and express interferon gamma-induced indoleamine-2,3 dioxygenase, a mediator of immunosuppression [26], they can survive for a long term in the homed tissue. In fact, intravenously administered allogenic Muse cells could survive for over 6 months in the host tissue without administering any immunosuppressants in preclinical studies [9, 24]. Based on these attractive properties, clinical trials for several diseases, including neonatal hypoxic–ischemic encephalopathy [30, 31], adult subacute stroke [32], acute myocardial infarction [33], adult epidermolysis bullosa [34], amyotrophic lateral sclerosis [35], and cervical spinal cord injury [36], that utilized the intravenous administration of donor-derived allogeneic Muse cells without HLA matching and immunosuppressant treatment have been initiated in Japan.

The effects of stem cell therapy for BPD to date were not derived from the replacement of the injured cells with exogenous MSCs, but from the paracrine effect by the trophic factors secreted from the stem cells [37]. If we can combine the replacement effect using Muse cells with the paracrine effect, we may achieve enhanced therapeutic outcomes. Even in terms of the paracrine effect, the ability of Muse cells to home into the injured sites offers a considerable advantage. In the present study, we evaluated the therapeutic effects of the intravenous administration of Muse cells in hyperoxia-induced lung injury model with features of bronchopulmonary dysplasia in rats and compared these outcomes with those of treatments using non-Muse MSCs (non-Muse cells).

In a previous study [37] on BPD using MSCs, the dosage, timing, and route of administration were considered important factors. Moreover, it has been reported that the intratracheal administration of MSCs is more effective than their intravenous administration for the treatment of BPD [38]. Therefore, in the present study, we aimed to compare the therapeutic effects between the intravenous and intratracheal administrations of Muse cells using a hyperoxia-induced neonatal lung injury in a rat model with features of bronchopulmonary dysplasia.

## Methods

All animal experiments were approved by the Nagoya University Animal Experiment Committee (Protocol Nos. 26126, 27190, 28215, 29012, 30197, 31106, 20032, and M210107-003) and conducted in accordance with the Regulations on Animal Experiments in Nagoya University. The present study complies with the ARRIVE guidelines (Animal Research: Reporting In Vivo Experiments). The experimental unit was the individual rat.

### Isolation of human Muse and non-Muse cells

Human muse and non-muse cells were isolated as described previously [22, 39]. Human bone marrow (BM)-MSCs (Lonza, Basel, Switzerland, PT-2501) were cultured at 37 °C, 5% CO2 in α-minimum essential medium (α-MEM; MilliporeSigma, St. Louis, MO, USA, M4526) containing 10% (vol/vol) fetal bovine serum (FBS; HyClone, Logan, UT, USA), 1 ng/mL basic fibroblast growth factor (Miltenyi Biotec, Auburn, CA, USA, 130-093-840), 2 mM GlutaMAX (GIBCO, Carlsbad, CA, USA, 35050-061) and 0.1 mg/mL kanamycin sulfate (GIBCO, 15160-054). Cells from passages 5 through 9 were used for Muse cell isolation. For preparation of green fluorescent protein (GFP)-labeled Muse and non-Muse cells, human BM-MSCs were introduced with vectors below. For lentivirus production, pMD2G, pCMV deltaR8.74, and pWPXL-GFP were transfected into LentiX-293T packaging cells (Takara Bio Inc, Shiga, Japan) using Lipofectamine 2000 (Thermo Fisher Scientific, MA, USA). After 3 days, the viral supernatant was collected, centrifuged, and filtered through a 0.45-μm filter. For collecting human Muse and non-Muse cells, GFP-labeled human BM-MSCs were incubated with anti-stage-specific embryonic antigen-3 (SSEA-3) antibody (1:1000; BioLegend, San Diego, CA, USA, 330302) followed by staining with a secondary antibody, allophycocyanin-conjugated anti-rat IgM (1:100; Jackson ImmunoResearch Laboratories, Inc., West Grove, PA, USA, 112-136-075). Cells were separated into SSEA-3+ (Muse) and SSEA-3- (non-Muse) fractions by fluorescence activated cell sorter (FACS; BD FACSAriaTM II cell sorter, Becton Dickinson, San Jose, CA, USA) from GFP-labeled human BM-MSCs. After cell sorting, the isolated Muse cells were cultured under adherent conditions for 24–48 h and then detached for administration.

### Animal models and experimental protocol

Pregnant Sprague–Dawley rats were obtained from Japan SLC Inc. (Shizuoka, Japan). They were healthy, non-genetically modified, and had not previously undergone any procedures. Pregnant rats at 16 days’ gestation were transported to the animal facility of Nagoya University and acclimatized until delivery on gestational day 21. The rats were housed under 12-h light/12-h dark cycles (9.00 A.M. to 9.00 P.M.) with the temperature controlled at 23 °C.

The pups were placed in an incubator within 24 h of birth and exposed to hyperoxia (83%), using a high/low oxygen control system for small animal experiments (BioSpherix, Parish, NY) until postnatal day 15. Muse (1 × 10^4^ cells; Muse group) or non-Muse cells (1 × 10^4^ cells; non-Muse group) in 0.1-mL acetic acid Ringer's solution or vehicle (only 0.1-mL acetic acid Ringer's solution: vehicle group) were administered slowly via the right external jugular vein or trachea on postnatal day 5. The rats in the sham group were housed under room-air conditions. The dams were rotated once every 2 days between the litters in the normoxia and hyperoxia groups to avoid excessive oxygen toxicity. The rats were sacrificed on day 15 or 29 to evaluate the treatment effect. On day 5, pups exposed to hyperoxia were allocated to the vehicle and cell-treated groups. In this neonatal model, pups were allocated to experimental groups while balancing body weight and litter distribution to minimize litter effects and baseline differences between groups. Within these constraints, pups were assigned randomly to each group. The group allocations and experimental procedures were not blinded. However, the evaluations of tissues and bronchoalveolar lavage fluid (BALF) were conducted in a manner blinded to group assignment.

The minimum sample for comparing the outcomes between the intravenous and intratracheal injections was calculated based on the preliminary experiments to achieve an 80% power of testing with an error rate of 1.67%, assuming a difference of 0.005 and standard deviation of 0.0065 in the tidal volume (TV) as a primary endpoint. The total sample size was calculated as n = 36. Additionally, in our previous experiments, approximately 20% of the intratracheal rats would occasionally die, particularly during the experimental process; therefore, the number of rats for evaluations was set to 44 per group. The minimum sample for comparing the outcomes between therapies using non-Muse and Muse cells was calculated based on the preliminary experiments to achieve an 80% power of testing with an error rate of 1.25%, assuming a difference of 0.025 and a standard deviation of 0.0020 in the TV as a primary endpoint. In this case, the total sample size was calculated as n = 15. Given that rats receiving intravenous injections rarely die, the number of rats for evaluations was set to 15 per group. Rats with TV values of >  + 4 SD were considered outliers and excluded from the analysis. Two rats were excluded.

The humane endpoints in this study were as follows: if severe lethargy (loss of spontaneous activity or inability to feed or drink) persisted or if the animals exhibited signs of severe distress, such as respiratory difficulty, abnormal posture, or convulsions, the experiment was terminated. Animals meeting these criteria were humanely euthanized by CO₂ inhalation. If any animals showed signs of unexpected suffering, they were immediately evaluated and euthanized according to these criteria.

### Body weight and survival rate

The rats’ body weights were evaluated from day 5 to day 15. The cumulative survival rate in each group was also evaluated every other day from birth to day 15.

### Respiratory function test: whole body plethysmography (WBP)

A respiratory function test was performed on day 15. TV was measured using WBP, which is an unrestrained respiratory function analyzer (IOX, emka TECHNOLOGIES Co., Ltd., Paris, France.). Each rat was housed in each animal chamber. After confirming that the respiratory parameters were stable, recording was started and continued for > 10 min. From the total respiratory data, a 2-min segment when the respiratory rate was stable was selected for analysis.

### Tissue preparation

The rats were deeply anesthetized with an intraperitoneal injection of overdosed pentobarbital or an anesthetic mixture comprising medetomidine, midazolam, and butorphanol[40] on day 29. Then, these rats were transcardially perfused with saline. The lungs were fixed using 4% paraformaldehyde via a tracheal catheter and kept at 20-cmH_2_O pressure for 20 min to be inflated, followed by immersion–fixation in 4% paraformaldehyde overnight at 4 °C. Subsequently, the right lungs were immersed in 20% and 30% sucrose solutions for 24 h each and embedded in a Tissue-Tek OCT solution (Sakura Finetek, Torrance, CA, USA) for the preparation of 30-µm frozen sections. Contrarily, the left lungs were dehydrated with a graded series of ethanol and xylene and embedded in paraffin for the preparation of 5-μm sections.

### Tissue morphometry

After deparaffinization and rehydration with xylene and graded alcohols to water, the paraffin sections were stained with hematoxylin and eosin (H&E). To assess the alveolar maldevelopment, the tissue volume densities (TVDs) in the left lungs were evaluated.

The TVD was evaluated as described previously [11]. With 100 evenly spaced points (a 10 × 10 grid), 300 µm apart, in each of the three random fields with six sections (a total of 18 fields), the proportion of lung tissue (alveolar ducts and sacs) in the lungs was evaluated using Stereo Investigator version 2020 stereology software (MicroBrightField, Williston, VT).

### Differential cell counts of bronchoalveolar lavage fluid

On day15, BALF was evaluated. The BALF was collected by instilling 0.6 mL (0.3 × 2) saline via a tracheal tube. After staining with Türk solution, the total cell number of BALF was counted using Burker chambers. Then, a 100-μL aliquot was centrifuged and plated onto the glass slides. Differential cell counts (macrophage, lymphocyte, neutrophil) were made, with at least 200 cells per animal, by staining with May–Giemsa [41, 42].

### Assessment of lung inflammation

Inflammatory macrophages in lung tissue were assessed by immunofluorescence staining for ED1 (CD68), a macrophage marker, and iNOS (inducible nitric oxide synthase), a marker of pro-inflammatory macrophage activation. Lung tissue samples were obtained from rats on postnatal day 15 and prepared using the same procedure as described above for tissue preparation. The lung sections were immunostained as previously described [11]. The following primary antibodies were used in immunofluorescence staining: mouse anti-ED1 (Abcam;diluted to 1:300), rabbit anti inducible nitric oxide synthase (iNOS)(Abcam;diluted to 1:40). Three random fields of 352 × 264 μm under 200 × magnification in two sections each were examined to quantify ED1- and iNOS-double-positive cells as inflammatory macrophages.

Serum TNF-α levels were measured using a Quantikine ELISA Rat TNF-α Immunoassay kit (RTA00-1; R&D Systems, Minneapolis, MN, USA) according to the manufacturer’s instructions. Serum samples were collected from rats on postnatal day 8.

### Quantitative polymerase chain reaction with the Alu sequence specific primer

To evaluate the distribution of Muse and non-Muse cells, quantitative polymerase chain reaction (qPCR) assays were performed with Alu elements, which are primate-specific repeats and comprise 11% of the human genome [43], as previously described [9]. Briefly, genomic DNA from the brain, lung, liver and spleen was collected after the intravenous administration of Muse or non-Muse cells on day 15. PCRs were performed with a volume of 20 μL, containing 10-μL TaqMan Universal Master Mix II (Applied Biosystems, Waltham, MA), 900-nM forward and reverse primers, 250-nM TaqMan probe, and 100-ng target template. Amplification was initiated at 50 °C for 2 min and 95 °C for 10 min, followed by 45 cycles of an incubation step at 95 °C for 15 s and 60 °C for 1 min.

### Immunofluorescence staining and image analysis

Free-floating lung sections (30 μm) were washed in PBS (3 × 10 min) and subjected to antigen retrieval in HistoVT One (Nacalai Tesque) at 70 °C for 30 min, followed by cooling to room temperature. Sections were blocked in PBS containing 10% normal donkey serum (IHR-8135, ImmunoBioScience) and 0.01% Triton X-100 (MP Biomedicals) for 1 h at room temperature. Unless otherwise stated, sections were washed in PBS containing 0.01% Triton X-100 (3 × 10 min) between incubations. Sections were incubated with goat anti-GFP (1:50, ab6673, Abcam) diluted in Can Get Signal Solution B (TOYOBO) for 24 h at 4 °C, followed by DyLight 488–conjugated donkey anti-goat IgG (H&L) (1:500, 605–741-002, Rockland) for 10 h at room temperature. Sections were then incubated (24 h, 4 °C) with one of the following primary antibodies diluted in Can Get Signal Solution B: mouse anti-STEM121 (1:50, Y40410, Takara Bio), mouse anti-hPE10 (1:50, 10375, IBL), rat anti-hPDPN (1:50, 018-24101, FUJIFILM Wako), rabbit anti-CTNNB1 (1:100, 51067-2-AP, Proteintech), or rabbit anti-ANXA1 (1:100, 21990-1-AP, Proteintech), followed by the corresponding DyLight 549–conjugated secondary antibodies (all 1:500, 10 h at room temperature; Rockland): donkey anti-mouse IgG (H&L) (610-742-124), rabbit anti-rat IgG (H&L) (612-442-026), or goat anti-rabbit IgG (H&L) (611-142-002). Nuclei were counterstained with Hoechst 33342 (10 μg/mL, Abcam). All steps were performed protected from light, and sections were mounted with ProLong Glass mounting medium.

Images were acquired using an LSM 880 confocal laser scanning microscope equipped with an Airyscan detector (Carl Zeiss Microscopy GmbH, Jena, Germany) controlled by ZEN software (Carl Zeiss Microscopy GmbH) under identical acquisition settings across groups. Quantification of GFP- or STEM121-positive area (mm^2^) normalized to lung tissue area (per 1 mm^2^) was performed in ZEN using an identical, predefined thresholding/segmentation procedure applied uniformly to all images by an investigator blinded to group allocation.

### Assessment of pulmonary hypertension

To assess for pulmonary hypertension, the dry weight ratio of the right ventricle (RV) to the left ventricle (LV) plus interventricular septum (IVS) (RV/LV + IVS) and right ventricular systolic pressure (RVSP) were used. The hearts obtained on day 29 were dissected, and the RV was separated from the LV and IVS. These tissues were dried up in a drying oven at 60 °C for 48 h. The weight of each tissue was measured, and the RV/LV + S ratio was calculated [44].

To measure the RVSP, the external jugular vein was exposed under isoflurane anesthesia, and a catheter (Natsume Seisakusho Co., Ltd. Tokyo, Japan) was inserted into the RV. The RVSP was measured by a pressure transducer and an amplifier system attached to the catheter (LEG-1000; Nihon Kohden Co, Tokyo, Japan) on day 29.

### Proteomics

#### Protein preparation

Lung tissues were collected from postnatal day-15 rats (n = 8/group) and processed based on our previous report [45] with the parameters described below. To avoid perfusion-related alterations in tissue proteome profiles, tissues for proteomics were collected without transcardial perfusion. Samples were snap-frozen in liquid nitrogen, pulverized using a Multi-beads shocker (Yasui Kikai Co., Ltd., Osaka, Japan), and homogenized in T-PER protein extraction reagent (20 mL/g tissue; Takara Bio Inc., Shiga, Japan) supplemented with an EDTA-free protease inhibitor cocktail (Complete; Roche Diagnostics, Basel, Switzerland). Homogenates were centrifuged at 11,000 × g for 15 min at 4 °C, and supernatants were collected. Detergents/surfactants were removed using Pierce Detergent Removal Spin Columns (Thermo Fisher Scientific, MA, USA), and total protein concentrations were determined by BCA assay (Pierce BCA Protein Assay Kit; Thermo Fisher Scientific). Protein extracts were adjusted to 100 µg/200 µL per sample and labeled with TMT 6-plex reagents (Thermo Fisher Scientific) for nanoLC–MS/MS.

The 32 samples (n = 8 per group) were analyzed in eight TMT 6-plex batches. In each batch, one biological sample from each experimental group (Sham, Vehicle, non-Muse, and Muse) and two pooled reference samples prepared by combining equal peptide aliquots from all study samples were included, with sample-to-channel assignment randomized across batches. Reporter-ion intensities were median-normalized within each plex and normalized across plexes by internal reference scaling using the pooled reference channels.

### Proteomic analysis

TMT-labeled samples were analyzed by nanoLC–MS/MS using an Orbitrap Fusion mass spectrometer (Thermo Fisher Scientific) coupled to an UltiMate 3000 RSLCnano LC system (Dionex Co., Amsterdam, The Netherlands) and a nanocapillary column (150 mm × 75 µm i.d.; Nikkyo Technos Co., Tokyo, Japan) via a nanoelectrospray ion source. Peptides were separated by reversed-phase chromatography at 300 nL/min using a linear gradient from 5 to 40% solvent B over 100 min (solvent A: 2% acetonitrile with 0.1% formic acid; solvent B: 95% acetonitrile with 0.1% formic acid). MS1 scans were acquired over m/z 400–1600. MS/MS was performed using quadrupole isolation (0.8 Th) and HCD fragmentation (30% normalized collision energy) with ion trap rapid scan readout. Precursors with charge states 2–6 were selected for MS/MS. Dynamic exclusion was set to 15 s with a 10-ppm tolerance, and the instrument was operated at maximum speed in 3-s cycles.

Peptide/protein identification and quantification were performed using Proteome Discoverer 1.4 (Thermo Fisher Scientific) with Mascot 2.6.0 (Matrix Science Inc., Boston, MA, USA) against the UniProt database (release 2021_01) using a precursor mass tolerance of 10 ppm and a fragment ion mass tolerance of 0.8 Da. Scaffold (Scaffold_4.4.8; Proteome Software Inc., Portland, OR, USA) was used to validate peptide and protein identifications. Raw data were deposited to jPOSTrepo (JPST003674) and ProteomeXchange (PXD061493).

### Analytical process and extraction criteria of the proteomic data

Protein abundances were compared among the four groups (sham, hyperoxia-induced lung injury model(hyperoxia)-vehicle, hyperoxia-Muse, and hyperoxia-non-Muse) using the Steel–Dwass nonparametric multiple comparison test. False discovery rate (FDR) was estimated using Storey’s method, and significance thresholds were set at *p* < 0.05 and FDR < 0.10. To disentangle disease- and treatment-associated changes, proteins were first classified as hyperoxia -affected or hyperoxia-unaffected based on sham-vehicle vs hyperoxia-vehicle. The sham-vehicle and hyperoxia-vehicle groups were compared to extract proteins affected or not by hyperoxia. The extracted proteins were divided into those showing significant effects from the Muse cell injection and those that did not, by comparing the hyperoxia-vehicle and hyperoxia-Muse groups. Then, by comparing the hyperoxia-vehicle and hyperoxia-non Muse groups, these proteins were further separated into those exhibiting significant effects from the non-Muse cell injections and those that did not, by comparing the hyperoxia-vehicle and hyperoxia-non-Muse groups.

The protein groups extracted by this stratified analysis were as follows (Supplemental Fig. 1a): proteins affected by hyperoxia (I); proteins not affected by hyperoxia (II); among the proteins affected by hyperoxia, those showing significant effects from the Muse cell injections (III); proteins that did not receive significant effects from the Muse cell injections (IV); proteins showing significant effects from therapy with MSCs, including Muse cells (V); proteins showing significant effects from treatment with only Muse cells (VI); proteins demonstrating significant effects from treatment with MSCs, not including Muse cells (VII); and proteins not showing significant effects from treatment with all MSCs, including Muse cells (VIII); among the proteins not affected by hyperoxia, the proteins showing significant effects from the treatment with Muse cells (IX); proteins not showing significant effects from the treatment with Muse cells (X); proteins not showing significant effects from the treatment with MSCs, including Muse cells (XI); proteins exhibiting significant effects from the treatment with only the Muse cells (XII); proteins showing significant effects from the treatment with MSCs, not including Muse cells (XIII); and Others. The enrichment ratio was defined as the ratio of the proportion of treatment-responsive proteins among hyperoxia -affected proteins to the proportion of treatment-responsive proteins among hyperoxia -unaffected proteins, i.e.,

$$ Enrichment \,ratio = \frac{{N_{\it{responsive,hyperoxia - affected}} /N_{\it{hyperoxia - affected}} }}{{N_{\it{responsive,hyperoxia - unaffected}} /N_{\it{hyperoxia - unaffected}} }} $$

### Assessment of potential confounding by residual intravascular blood

Because residual intravascular blood can contribute erythrocyte-, platelet-, and plasma-derived proteins to lung tissue lysates used for proteomics, we quantified erythrocyte-enriched marker proteins (hemoglobin subunits α/β, carbonic anhydrase 1, band 3/SLC4A1, glycophorin C, and peroxiredoxin-2) and a platelet-enriched marker protein (platelet factor 4, PF4). [45] For each sample *i*, we calculated an erythrocyte marker index (EMI) and a PF4 index as percentages of the total protein abundance:

1. $${A}_{i,Total}=\sum_{q\in P}{A}_{i,q}$$
2. $${EMI}_{i}=100\times \frac{\sum_{p\in R}{A}_{i,p}}{{A}_{i,Total}}$$
3. $${PF4}_{i}=100\times \frac{{A}_{i,PF4}}{{A}_{i,Total}}$$

where *A*_*i,p*_ denotes the LC–MS/MS-derived abundance of protein *p* in sample *i* (*i* indexes samples; *p* and* q* index proteins), *R* is the set of erythrocyte marker proteins (*R* = {Hba, Hbb1, Hbb2, Ca1, Slc4a1, Gypc, Prdx2}), and ${A}_{i,Total}=\sum_{q\in P}{A}_{i,q}$ is the total abundance summed over all quantified proteins *P* in sample *i*. In addition, we derived the hemoglobin fraction (Hb fraction), a coagulation score (Fga + Fgb + Fgg + F2), and a plasma proxy index (Alb + Fga + Fgb + Fgg + F2 + C3 + C4 + C9) as %Total. Protein abundances reported as not detected (“n.d.”; below the quantification limit) were imputed with a fixed quantification-limit value (55,671.01) defined as the minimum quantifiable non-zero abundance observed in the dataset prior to calculation [46, 47]. *Hb*_*sum*_ and *Coag*_*sum*_ did not require imputation because all component proteins were quantified in all samples; imputation was applied only for RBC-markersum and PF4 calculations.

To test whether the coagulation/fibrinolysis-related signal could be explained by sample-to-sample variability in residual blood, we assessed the association between the summed raw abundance of hemoglobin subunits and the summed raw abundance of core coagulation proteins across all samples using Spearman’s rank correlation. Specifically:

(4) $${Hb}_{sum,i}={A}_{i,Hba}+{A}_{i,Hbb1}+{A}_{i,Hbb2}$$   
(5) $${Coag}_{sum,i}={A}_{i,Fga}+{A}_{i,Fgb}+{A}_{i,Fgg}+{A}_{i,F2}$$    

Spearman rs and two-sided p-values were reported [48].

### Functional analysis

In the protein profiles extracted by these 13 conditions, groups I (proteins affected by hyperoxia), III (among the proteins affected by hyperoxia, those showing significant effects from the treatment with only Muse cell injections), and V + VII (among the proteins affected by hyperoxia, those showing significant effects from the treatment with non-Muse cell injections) were used for the functional annotation analysis on the DAVID 6.8 (database accessed Feb 19, 2021) (Huang et al., 2009). Functional annotation clustering was performed to enrich the proteins of these 12 protein profiles into gene ontology (GO term). The flagged GO terms were then clustered to clarify the relationships among reported annotation and co-association proteins. Among the clustered GO terms, we extracted the groups with the top 5 cluster enrichment scores.

Protein–protein interaction networks were visualized using the STRING v11.0. and clustered using Markov cluster algorithm (MCL; inflation parameter = 3) with a minimum required interaction score of 0.400. The interactions were indicated by eight criteria for linkage, including neighborhood, gene fusion, co-occurrence, co-expression, experiments, databases, text mining, and homology. Information on the distribution and main function of the extracted proteins in the lung was also investigated using the Human Protein Atlas (https://www.proteinatlas.org/) and UniProt (https://www.uniprot.org/) databases.

### Statistical analyses

Statistical analyses were performed using the Prism 10 software (GraphPad Software, Boston, MA). The survival rate was evaluated by using the Kaplan–Meier method. Proteomic analysis was evaluated by using the Steel–Dwass test. For analyses of TV, TVD, and BALF, which involved two independent variables (route of administration and treatment), two-way analysis of variance was used, followed by post hoc multiple comparison tests when appropriate. The other analyses were performed using one-way analysis of variance, followed by the Holm Sidak method. Spearman’s rank correlation coefficient was used to assess associations between summed raw abundances of hemoglobin subunits and coagulation proteins across samples. All values were presented as the mean with standard error. Statistical significance was defined as *p* < 0.05.

## Results

### Comparison between the intravenous and intratracheal administrations

#### Body weight and survival rate

**Fig. 1:**
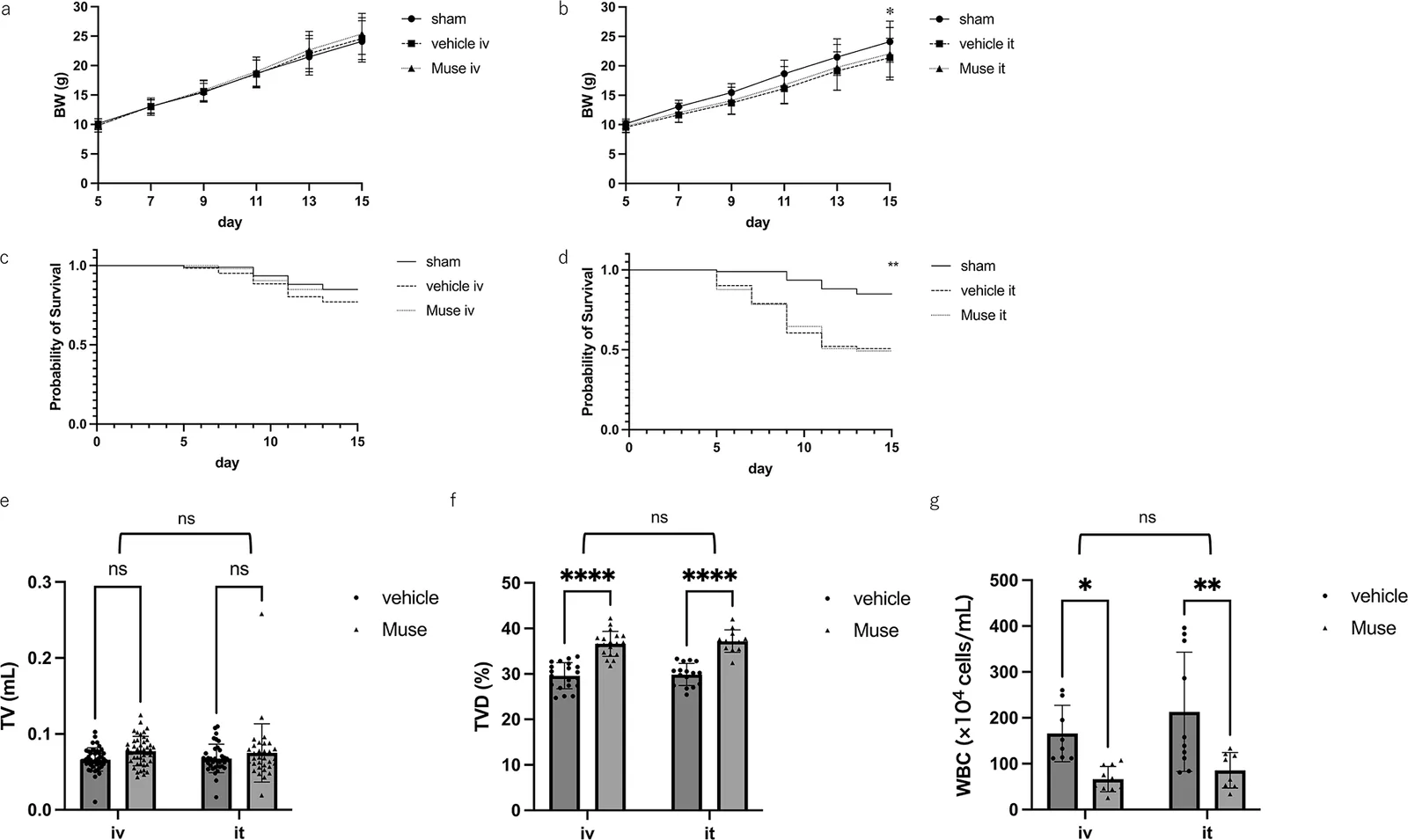
Comparison between intravenous and intratracheal administration of Muse cells in hyperoxia-induced lung injury model. **a** Body weight in the intravenous (iv) groups was no significant difference among the groups. (n = 51 for sham, 43 for vehicle, and 44 for Muse). **b** Body weight in the intratracheal (it) groups. Regardless of whether the cells were administered or not, there was a reduction in weight gain. (n = 51 for sham, 36 for vehicle, and 32 for Muse). *p < 0.05; sham vs vehicle-it and Muse-it. **c** Survival rate in the iv groups. No significant difference was noted among the groups (survive on day 15; n = 51 for sham, 43 for vehicle, and 44 for Muse). **d** Survival rate in the it group. Regardless of whether the cells were administered or not, a worsening of the survival rate was noted in the it groups. (survive on day15; n = 51 for sham, 36 for vehicle, and 32 for Muse). **p < 0.01; sham vs vehicle-it and Muse-it. **e** For TV on day 15, there were no significant main effects of route of administration or treatment, nor was there a significant interaction (n = 43 for vehicle-iv, 44 for Muse-iv, 36 for vehicle-it, and 32 for Muse-it). **f** For TVD on day 29, Muse treatment significantly improved TVD compared with the vehicle in both the IV and IT groups (*p* < 0.0001 for both), whereas no significant difference was observed between the routes of administration (n = 20 for vehicle-iv, 19 for Muse-iv, 16 for vehicle-it, 12 for Muse-it). ****p < 0.0001. **g** Muse treatment significantly improved BALF WBC counts compared with the vehicle in both the iv and it groups (*p* < 0.05 for iv, *p* < 0.01 for it), whereas no significant difference was observed between the routes of administration. (n = 8 for vehicle-iv, 10 for Muse-iv, 10 for vehicle-it, 8 for Muse-it)

In the intravenous (iv) group, the body weight on day 15 and survival rate were not significantly different among the sham, vehicle, and Muse groups (Fig. 1a, c). However, in the intratracheal (it) groups, regardless of whether the cells were administered or not, a slowing in weight gain (*p* < 0.05; sham vs vehicle-it and Muse-it) and worsening of survival rate (*p* < 0.01; sham vs vehicle-it and Muse-it) were observed in the it groups than in the sham group (Fig. 1b, d).

### Respiratory function test: WBP

There were no significant main effects of route of administration or treatment, and no significant interaction. Although TV appeared higher in the iv Muse group than in the iv vehicle group, this difference did not reach statistical significance in post hoc multiple comparisons (Fig. 1e).

### Impact of Muse cells on the lung tissues

Muse treatment significantly improved TVD compared with vehicle in both iv and it groups (*p* < 0.0001), whereas no significant difference was observed between administration routes (Fig. 1f).

### Differential cell counts BALF

Muse treatment significantly improved BALF WBC counts compared with the vehicle in both the iv and it groups (*p* < 0.05 for iv, *p* < 0.01 for it), whereas no significant difference was observed between the routes of administration (Fig. 1g).

### Comparison of the intravenous Muse and non-Muse cell administrations with vehicle

#### Respiratory function test: WBP

**Fig. 2:**
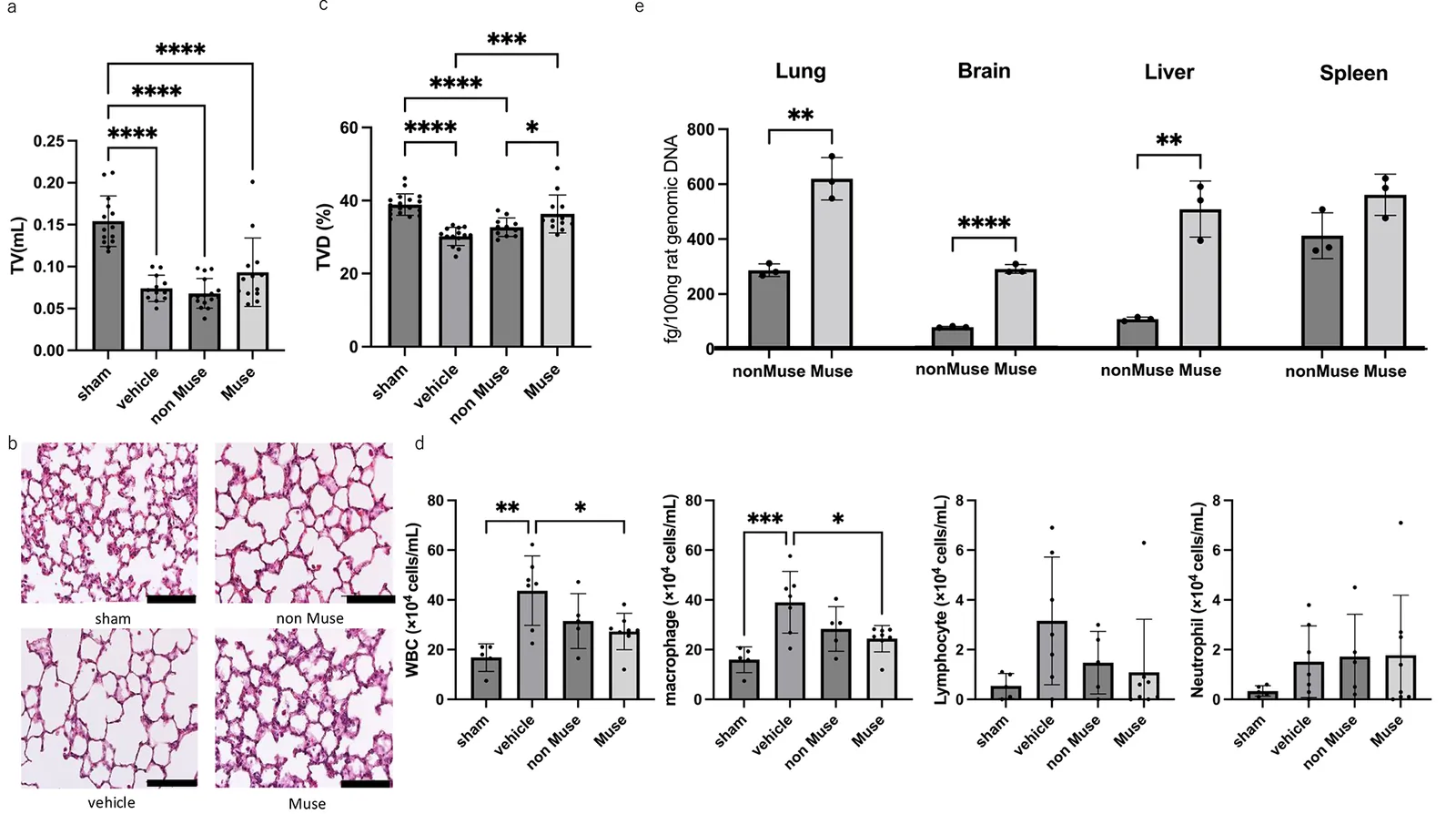
Comparison between intravenous administration of Muse and non-Muse cells in hyperoxia-induced lung injury rat model. **a** TV on day 15 was significantly lower in the vehicle group than in the sham group. However, no significant difference in TV was noted between the vehicle, non-Muse, and Muse groups. (n = 14 for sham, 12 for vehicle, 16 for non-Muse, and 13 for Muse). ****p < 0.0001. **b** Representative images of the alveolar spaces in the lung (H&E staining). Bar = 50 µm. **c** TVD on day 29 was significantly lower in the vehicle group than in the sham group and was significantly higher in the Muse group than in the vehicle and non-Muse groups. (n = 18 for sham, 14 for vehicle, 11 for non-Muse, and 12 for Muse). *p < 0.05, ***p < 0.001, ****p < 0.0001. **d** The WBC and Macrophage counts in BALF on day15 were significantly lower in the Muse group than in the vehicle group, but not significantly lower in the non-Muse group. Lymphocyte counts did not differ significantly among the groups. (n = 5 for sham, 7 for vehicle, 5 for non-Muse, and 8 for Muse). *p < 0.05, **p < 0.01, ***p < 0.001. **e** Amounts of DNA in most organs on day 15 were higher in the Muse group than in the non-Muse group. (n = 3 for non-Muse and 3 for Muse). *p < 0.05, **p < 0.01

The TV on day 15 was significantly lower in the vehicle group than in the sham (*P* < 0.0001). However, there is no significant difference between the vehicle, non-Muse, and Muse groups (Fig. 2a). The TV in the Muse group tended to be higher than that in the non-Muse group, although the difference is not significant (*p* = 0.053).

### Impact of Muse cells on lung tissue

Representative photographs on day 29 are shown in Fig. 2b. The alveolar spaces of the hyperoxia-loaded rats (vehicle) were more enlarged than those of the sham group, and this enlargement was ameliorated in the Muse group, whereas, in the non-Muse group, the improvement was poor. TVD was significantly lower in the vehicle group than in the sham group, and was significantly higher in the Muse group than in the vehicle and non-Muse groups on day 29 (Fig. 2c).

### Differential cell counts BALF

We evaluated the total and differential cell counts of BALF on day 15. The total number of white blood cells in BALF was increased in the vehicle group and was significantly reduced in the Muse group, but not in the non-Muse group, compared with the vehicle group. Differential cell counts based on May–Giemsa staining revealed that macrophages were the predominant cell type and that their absolute numbers were increased in the vehicle group and significantly attenuated in the Muse group. In contrast, the absolute numbers of lymphocytes and neutrophils did not differ significantly among the groups. (Fig. 2d).

### Assessment of lung inflammation

The number of ED1^+^ iNOS^+^ cell in the hyperoxia rats were elevated in the vehicle group compared with the sham group, and this increase was ameliorated by Muse cell administration (Supplemental Fig. 2a). Serum TNF-α levels in the hyperoxia rats were elevated in the vehicle group compared with the sham group, and this increase was ameliorated by Muse cell administration (Supplemental Fig. 2b).

### Distribution of Muse and non-Muse cells after intravenous administration in hyperoxia-induced lung injury model rats

To evaluate the distribution of Muse and non-Muse cells, qPCR assay with human Alu elements (Alu PCR) was performed at 10 days after the intravenous administration (day 15). Alu PCR detected human genomic DNA in all of the organs (left lung, brain, liver, and spleen), with the highest amount being found in the lung of the Muse-treated rats. Human DNA was also detected in all of the organs of the non-Muse group. However, the amount of DNA in most organs was significantly lesser than that of the Muse group (Fig. 2e).

### Engraftment of Muse and non-Muse cells in the lung

**Fig. 3:**
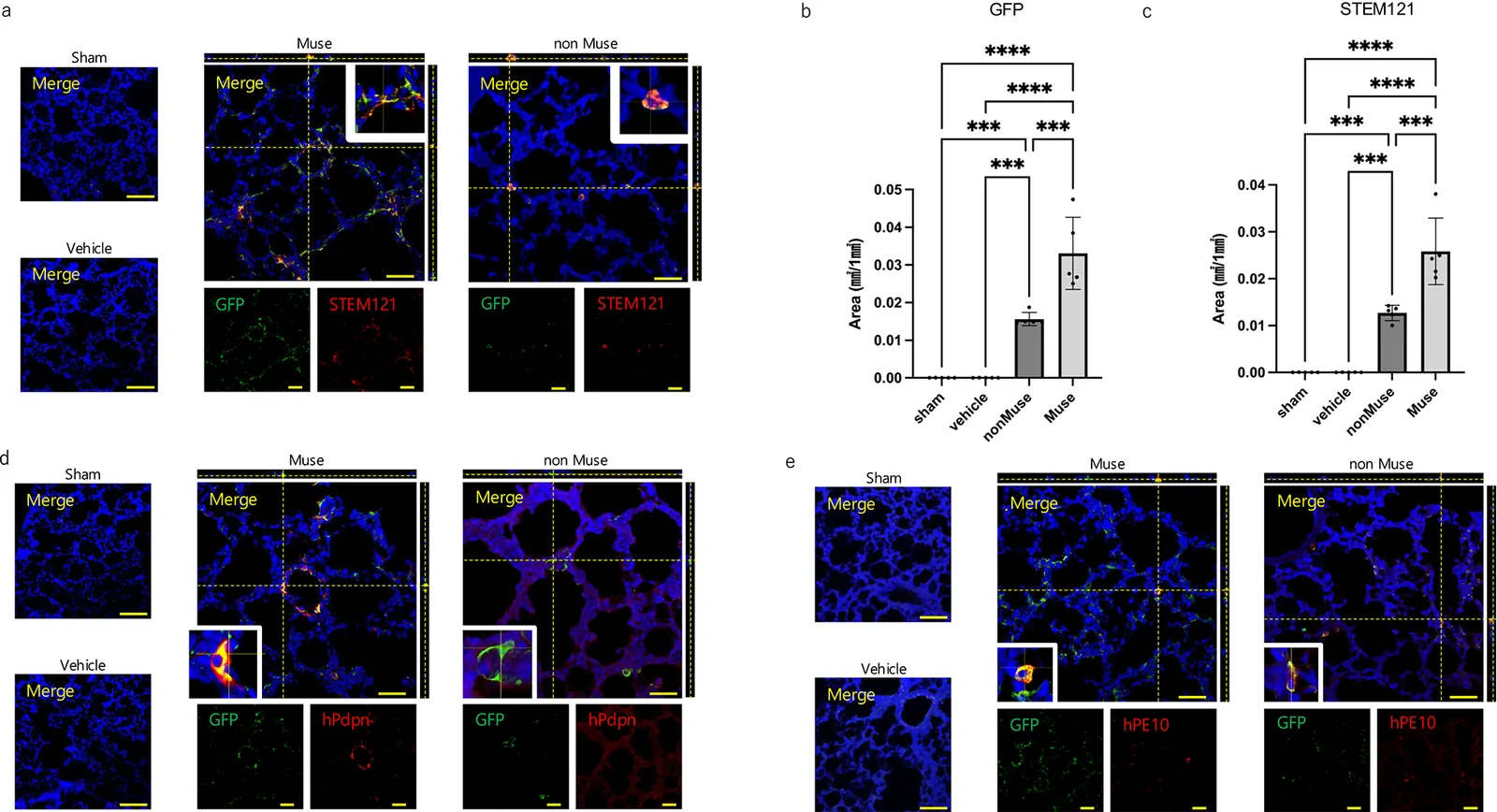
Immunofluorescence staining. **a** Immunofluorescence staining for GFP or STEM121. GFP- or STEM121-positive cells indicate engrafted Muse or non-Muse cells. The Muse group shows more flattened cells, whereas the non-Muse group showed more spherical cells. **b** Area of the GFP-positive cells per 1-mm^2^ lung tissue. The GFP-positive cells were detected in both the non-Muse and Muse groups, but the amount of these cells was significantly higher in the Muse group. ***p < 0.001, ****p < 0.0001. **c** Area of STEM121-positive cells per 1-mm^2^ lung tissue. The STEM121 positive cells were detected in both the non-Muse and Muse-treated groups, but the amount of these cells was significantly higher in the Muse group. ***p < 0.001, ****p < 0.0001. **d** Immunofluorescence staining for GFP or human podoplanin (hPdpn). Expression of human podoplanin, a marker of human type I alveolar epithelial cells, was confirmed in the GFP-positive cells in the GFP-labeled Muse cells. **e** Immunofluorescence staining for GFP or hPE10. Expression of human hPE10, a marker of human type2 alveolar epithelial cells, was confirmed in the GFP-positive cells in the GFP-labeled Muse cells

To confirm the engraftment of the Muse and non-Muse cells in the lung, immunofluorescence staining for GFP or STEM121 (human cell marker) was performed on day15. Figure 3a shows the representative pictures. The GFP or STEM121-positive cells indicated the presence of engrafted Muse or non-Muse cells. The Muse group showed more flattened cells, whereas the non-Muse group showed more spherical cells. Cells were detected in both the non-Muse group and Muse groups, but the number of cells was significantly higher in the Muse group in both markers (Fig. 3b, c).

### Differentiation of Muse cells into type 1 and 2 alveolar epithelial cells

Expression of human podoplanin, a marker of human type I alveolar epithelial cells, was confirmed in the GFP-positive squamous cells in the GFP-labeled Muse cells. The human podoplanin-positive cells were observed to localize in a ring-like pattern, (like an alveolar structure) (Fig. 3d). The expression of human hPE10, a marker of human type2 alveolar epithelial cells, was also confirmed in the GFP-positive cells in the GFP-labeled Muse cells (Fig. 3e).

### Assessment of pulmonary hypertension

**Fig. 4:**
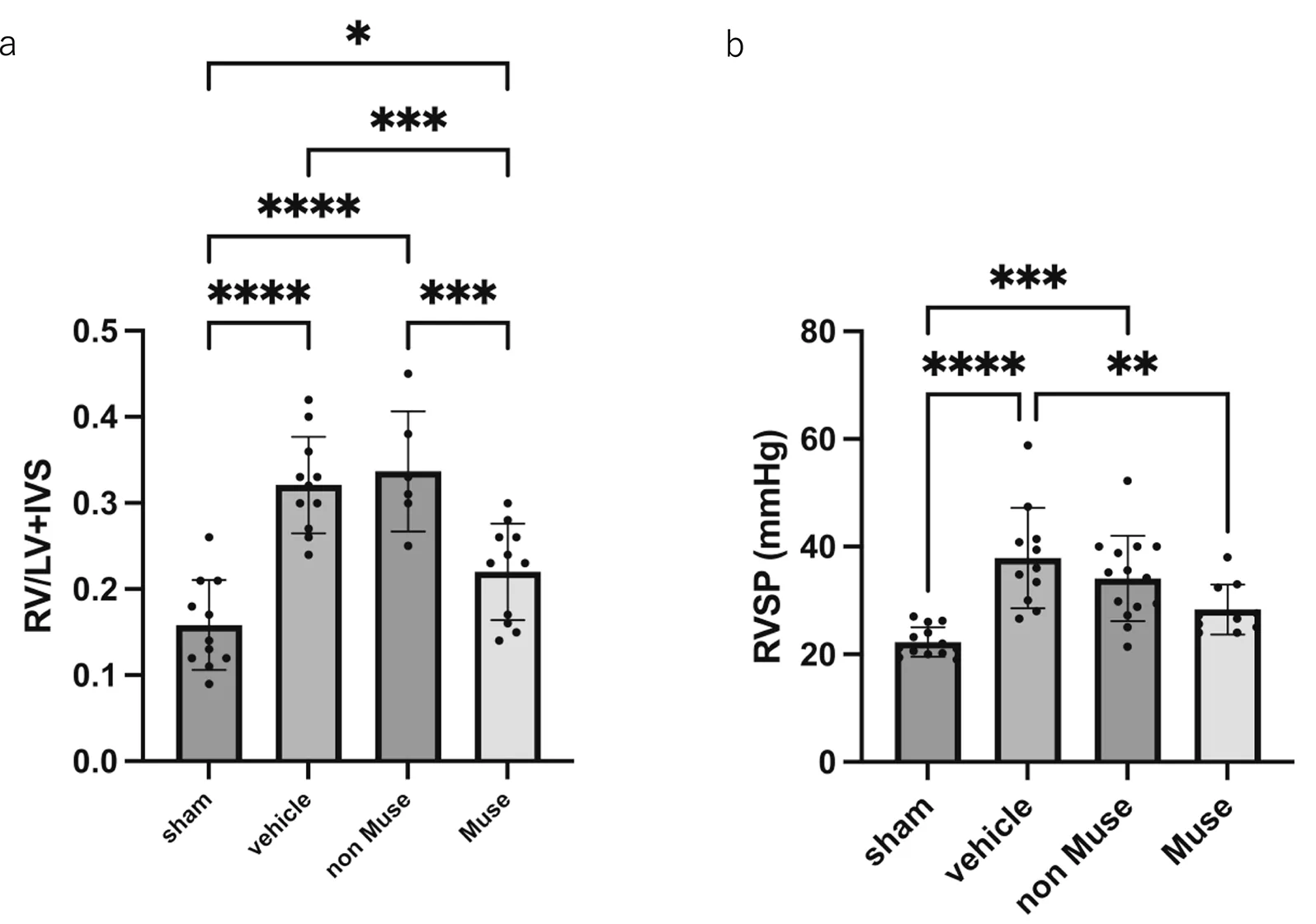
Evaluation of pulmonary hypertension. **a** RV/LV + S on day 29 was significantly lower in the Muse group than in the vehicle and non-Muse groups. (n = 11 for sham, 11 for vehicle, 6 for non-Muse, and 11 for Muse). *p < 0.05, ***p < 0.001, ****p < 0.0001. **b** RVSP on day 29 was significantly lower in the Muse group than in the vehicle group. (n = 13 for sham, 11 for vehicle, 14 for non-Muse, and 10 for Muse) **p < 0.01, ***p < 0.001, ****p < 0.0001

In hyperoxia-induced lung injury model, RV hypertrophy develops, evidenced by the elevated dry weight ratio of the RV to the LV plus IVS (RV/LV + IVS). Compared to the vehicle and non-Muse groups, the Muse group showed a significant decrease in the RV/LV + IVS on day 29 (*p* < 0.001) (Fig. 4a).

In addition, RV systolic pressure (RVSP) is increased in hyperoxia exposed groups. As compared to the sham group, the RVSP in the vehicle group was significantly higher at P29 (*p* < 0.0001). As compared to the vehicle group, the RVSP in the Muse group was significantly lower on day 29 (*p* < 0.01), (Fig. 4b). RV/LV + IVS showed a strong correlation with RVSP (r = 0.78, *p* < 0.0001) (Supplemental Fig. 4).

### Proteomics

The protein profiles of the lungs obtained from the four experimental groups (sham, vehicle, Muse, and non-Muse groups) were analyzed to investigate the target molecules associated with the mechanisms of abnormal respiratory function and the therapeutic effects of the cells (Supplemental Fig. 1a, b). Using LC/MS/MS, 2694 proteins with high-quality signals were detected and quantified; of these, 844 proteins exhibited significant differences between the Sham and Vehicle groups. Of the 844 proteins, 141 proteins were extracted as having been affected by the Muse cell administration and 703 proteins were not significantly affected by its administration. Furthermore, among the 141 proteins extracted, 56 showed significant effects not only from the treatment with Muse cells but also from the treatment with non-Muse cells. In other words, 85 proteins showed significant effects from the treatment with only the Muse cells. Contrarily, 167 proteins were significantly affected by the treatment with only the non-Muse cells. Of the 1850 proteins, not affected by hyperoxia, 115 and 227 proteins were significantly affected from the treatments with Muse and non-Muse cells, respectively; of these, 39 proteins were common between the Muse and non-Muse groups. According to the number of extracted proteins, the enrichment ratio of the proteins affected by Muse cell administration was 2.69, whereas that of the proteins affected by non-Muse cell administration was 2.15 (Supplemental Fig. 1c).

### Functional analysis

To understand the biological significance of each extracted protein profile, we performed a functional analysis using the GO term database and its clustering analysis. First, the proteins categorized as Group I, which includes 844 proteins affected by hyperoxia, were evaluated. As top five clusters, cell adhesion-related, ATP synthase-related, cAMP-dependent protein kinase-related, blood coagulation/fibrinolytic system-related, and lipoprotein-related ontologies were extracted through the analysis (Table 1).

**Table 1:** Top five clusters of significantly enriched GO terms in proteins affected by hyperoxia

Cluster	Enrichment score	Term name	*p*-value	Fold enrichment
Cluster 1	14.46	Cadherin binding involved in cell–cell adhesion	< 0.001	4.28
		Cell–cell adherens junction	< 0.001	4.07
		Cell–cell adhesion	< 0.001	3.97
Cluster 2	2.37	Proton-transporting ATP synthase complex, catalytic core F(1)	0.0016	14.97
		Mitochondrial proton-transporting ATP synthase complex, catalytic core F(1)	0.0016	14.97
		Mitochondrial proton-transporting ATP synthase complex	0.0024	6.12
		Proton-transporting ATP synthase activity, rotational mechanism	0.0099	5.69
		ATP synthesis coupled proton transport	0.024	4.47
Cluster 3	2.13	cAMP-dependent protein kinase regulator activity	0.0021	13.65
		cAMP-dependent protein kinase complex	0.0027	12.83
		Negative regulation of cAMP-dependent protein kinase activity	0.0047	10.73
		cAMP-dependent protein kinase inhibitor activity	0.0054	10.24
		Protein kinase A catalytic subunit binding	0.034	5.46
		cAMP binding	0.036	3.94
Cluster 4	1.64	Plasminogen activation	0.0094	8.59
		Positive regulation of heterotypic cell–cell adhesion	0.0094	8.59
		Protein polymerization	0.013	7.8
		Blood coagulation, fibrin clot formation	0.02	12.88
		Platelet alpha granule	0.027	5.99
		Fibrinogen complex	0.036	9.62
		Cellular protein complex assembly	0.056	4.52
		Positive regulation of peptide hormone secretion	0.063	7.15
Cluster 5	1.62	High-density lipoprotein particle assembly	0.0047	10.73
		Discoidal high-density lipoprotein particle	0.0058	22.45
		Reverse cholesterol transport	0.016	7.15
		High-density lipoprotein particle remodeling	0.1	5.37
		Lipid transporter activity	0.16	4.1

The proteins affected by hyperoxia were classified as Group III, which includes 141 proteins affected by Muse cell administration, and as Group V + VII, which includes 223 proteins affected by non-Muse cell administration. The analysis identified endopeptidase and inflammation-related, filament and cytoskeleton-related, blood coagulation/fibrinolytic system-related, and cell adhesion-related ontologies as the top five clusters of Group III (Table 2), whereas, for Group V + VII, these were GTPase-related, ribosome-related, filament-related, cytoskeleton-related, and hydrogen peroxide-related ontologies (Table 3).

**Table 2:** Top five enriched GO clusters in hyperoxia-related proteins affected by Muse cells

Cluster	Enrichment score	Term name	*p*-value	Fold enrichment
Cluster 1	3.04	Negative regulation of endopeptidase activity	< 0.001	6.82
		Endopeptidase inhibitor activity	0.0018	16.2
		Inflammatory response	0.0024	3.82
Cluster 2	2.49	Brush border	< 0.001	14.4
		Stress fiber	0.0018	9.65
		Calmodulin binding	0.04	3.87
		Myosin complex	0.055	7.84
		Motor activity	0.072	6.75
Cluster 3	2.2	Negative regulation of endopeptidase activity	< 0.001	6.82
		Vasodilation	0.001	20.18
		Negative regulation of blood coagulation	0.0039	31.54
		Cysteine-type endopeptidase inhibitor activity	0.048	8.48
		Positive regulation of cytosolic calcium ion concentration	0.35	2.43
Cluster 4	2.09	Stress fiber	0.0018	9.65
		Actin filament binding	0.0057	5.25
		Actin cytoskeleton	0.054	3.51
Cluster 5	2.07	Cadherin binding involved in cell–cell adhesion	0.0022	4.42
		Cell–cell adherens junction	0.0033	4.12
		Cell–cell adhesion	0.085	2.99

**Table 3:** Top five enriched GO clusters in hyperoxia-related proteins affected by non-Muse cells

Cluster	Enrichment score	Term name	*p*-value	Fold enrichment
Cluster 1	7.18	Small GTPase mediated signal transduction	< 0.001	6.19
		GTP binding	< 0.001	4.55
		GTPase activity	< 0.001	5.95
		GDP binding	< 0.001	11.78
Cluster 2	3.25	Cytosolic small ribosomal subunit	< 0.001	9.53
		Translation	0.0027	2.77
		Structural constituent of ribosome	0.0076	2.43
		Ribosome	0.098	2.85
Cluster 3	2.41	Intermediate filament	0.0013	7.39
		Type III intermediate filament	0.0013	50.97
		Structural molecule activity	0.034	2.91
Cluster 4	2.07	Structural constituent of cytoskeleton	< 0.001	7.64
		Microtubule-based process	0.014	7.85
		Cytoskeleton organization	0.022	3.77
		Microtubule	0.06	2.52
Cluster 5	1.91	Haptoglobin-hemoglobin complex	< 0.001	63.72
		Response to hydrogen peroxide	0.016	5.16
		Positive regulation of cell death	0.14	4.47

Notably, Cell adhesion-related ontologies were also commonly enriched as cluster 1 of Group I (hyperoxia.-affected proteins) and as cluster 5 of Group III (hyperoxia.-affected proteins responsive to Muse). Blood coagulation/fibrinolytic system-related ontologies were commonly enriched as clusters 4 and 3 of Groups I and III, respectively. These ontologies were not enriched in Group V + VII (non-Muse–responsive proteins). Moreover, filament and cytoskeleton-related ontologies were commonly enriched as clusters 2 and 4 of Group III and as clusters 3 and 4 of Group V + VII.

**Fig. 5:**
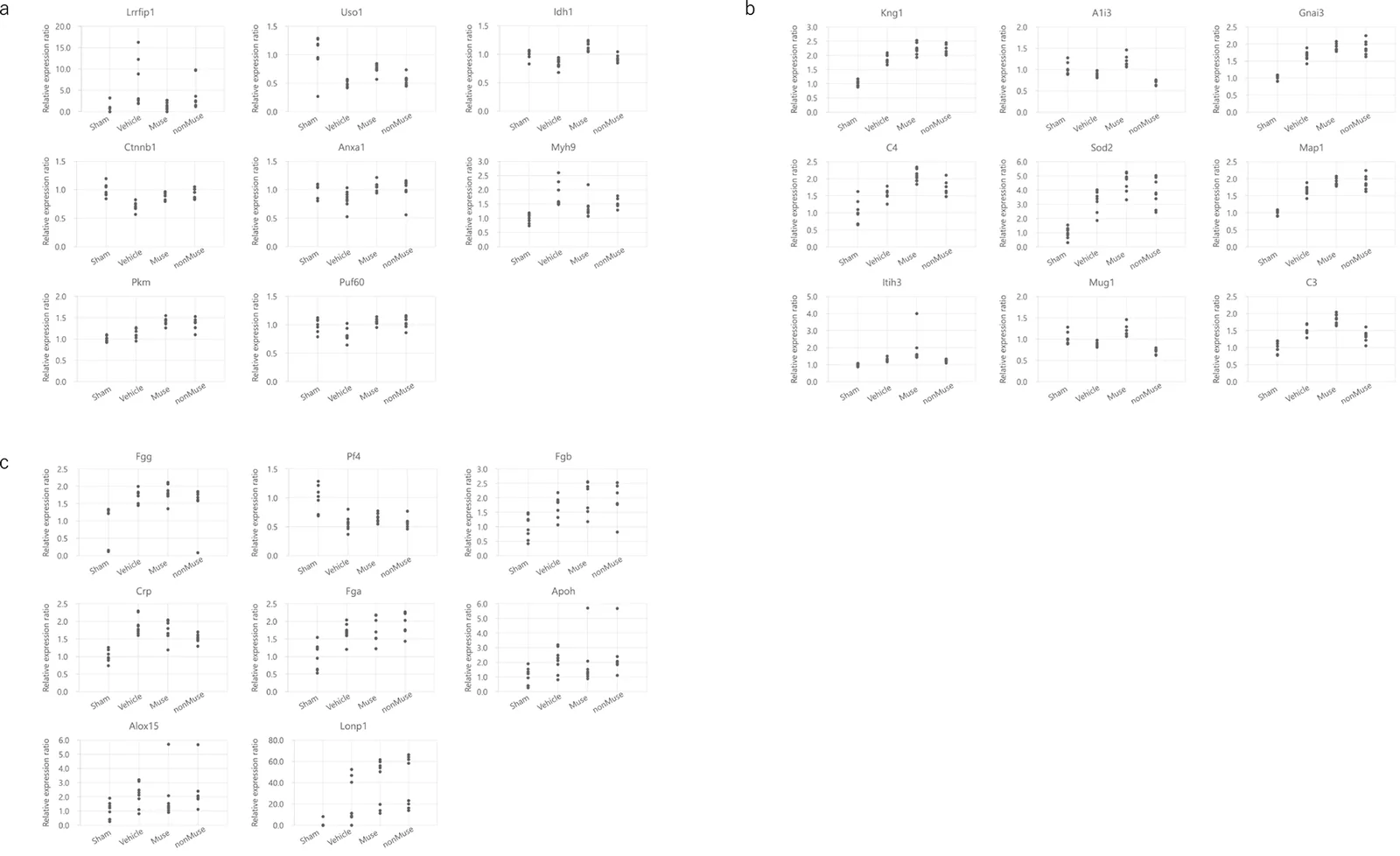
Expression levels of proteins in the ontologies extracted by the proteomic and functional analyses. **a** Expression levels of eight proteins included in the cell adhesion-related ontologies. Lrrfip1 and Myh9 were upregulated by hyperoxia and suppressed by Muse cell administration. The expressions of Uso1, Idh1, Ctnnb1, Anxa1, and Puf60 were downregulated by hyperoxia and subsequently upregulated by Muse cell administration. **b** Expression levels of proteins in the blood coagulation/fibrinolytic system-related ontologies affected by Muse cell administration. The expressions of A1i3 and Mug1 were downregulated by hyperoxia and upregulated by Muse cell administration, whereas the others were upregulated by hyperoxia and further upregulated by Muse cell administration. **c** The expression levels of proteins in the blood coagulation/fibrinolytic system-related ontologies not affected by Muse cell administration. Except for Pf4, these proteins were upregulated by hyperoxia. Dot plots show the expression levels of each protein in the four experimental groups (sham, vehicle, Muse, and non-Muse)

For the cell adhesion-related ontologies, eight proteins significantly affected by Muse cell administration were included. The distribution and main function of the eight proteins in the normal lungs are listed in Supplemental Table 1. The expression of all of the eight proteins was detected in the alveolar cells, bronchus, and macrophages in the normal lungs, indicating that their expression levels were altered by hyperoxia and Muse cells administration. Lrrfip1 and Myh9 were upregulated by hyperoxia and suppressed by Muse cell administration (Fig. 5a). Uso1, Idh1, Ctnnb1, Axa1, and Puf60 were downregulated by hyperoxia and enhanced by Muse cell administration (Fig. 5a). Finally, Pkm was upregulated by hyperoxia and further increased by Muse cell administration (Fig. 5a).

In contrast to the proteins with cell adhesion-related ontologies, almost all of proteins with blood coagulation/fibrinolytic system-related ontologies were not detected in the normal lungs (Supplemental Tables 2 and 3); hence, these proteins may be expressed and/or translocated by hyperoxia. The expressions of Gnai3, Sod2, and Map1, which are detected in the normal lungs, were upregulated by hyperoxia and further increased by Muse cell administration (Fig. 5b). Kng1, Itih3, C3, and C4, which are not detected in the normal lungs, were detected in the hyperoxia lungs and further increased by Muse cell administration (Fig. 5b). A1i3 and Mug1 were suppressed by hyperoxia and enhanced by Muse cell administration (Fig. 5b). Regarding the blood coagulation/fibrinolytic system-related proteins not affected by Muse cell administration, Alox15 and Lonp1 were detected in the normal lungs, whereas Fga, Fgb, Fgg, Apoh, Crp, and Pf4 were not detected in the normal lungs. The expression of every protein, except for Pf4, was increased by hyperoxia (Fig. 5c).

### Assessment of blood-derived signatures

To evaluate whether the coagulation/fibrinolysis-related signal could be confounded by variable residual intravascular blood, we assessed erythrocyte- and platelet-enriched marker proteins (Supplemental Table 4). The erythrocyte marker index (EMI; hemoglobin subunits α/β, carbonic anhydrase 1, SLC4A1, glycophorin C, and peroxiredoxin-2; expressed as % of total protein abundance) was not increased in the hyperoxia-exposed (Vehicle, non-Muse, and Muse) groups and was highest in Sham (Table 4). PF4, a platelet-enriched marker, showed a similar pattern. In contrast, the coagulation score (Fga + Fgb + Fgg + F2; % of total protein abundance) was higher in hyperoxia-exposed groups than in Sham, resulting in an increased coagulation-to-hemoglobin ratio (Table 4). The plasma proxy index was comparable across groups (Table 4).

**Table 4:** Blood-derived signature indices in lung proteomes

	Sham	Vehicle	Non-Muse	Muse
EMI (%Total)	25.3 ± 3.4	18.1 ± 1.7	14.8 ± 1.3	16.8 ± 1.1
Hemoglobin fraction (%Total)	24.5 ± 3.4	17.1 ± 1.8	14.4 ± 1.3	16.4 ± 1.1
PF4 (× 10^–3^%Total)	5.2 ± 1.1	2.9 ± 0.7	3.0 ± 0.5	3.5 ± 0.4
Coagulation (%Total)	0.44 ± 0.17	0.75 ± 0.08	0.69 ± 0.22	0.79 ± 0.10
Plasma proxy (%Total)	8.70 ± 0.88	9.36 ± 0.62	8.77 ± 0.7	9.60 ± 0.82
Coagulation/hemoglobin (ratio)	0.019 ± 0.009	0.043 ± 0.007	0.047 ± 0.014	0.048 ± 0.006

Importantly, across all samples (n = 32), the summed raw abundance of hemoglobin subunits (*Hb*_*sum*_; Hba + Hbb1 + Hbb2) showed no association with the summed raw abundance of core coagulation proteins (*Coag*_*sum*_; Fga + Fgb + Fgg + F2) (Spearman rs = 0.014, p = 0.94; Supplemental Fig. 3).

Erythrocyte marker index (EMI, %Total) was calculated as $100\times \frac{\sum_{p\in R}{A}_{i,\mathrm{p}}}{{A}_{i,Total}}$, where *R* = {Hba, Hbb1, Hbb2, Ca1, Slc4a1, Gypc, Prdx2}. Hemoglobin fraction (%Total) was calculated as $100\times \frac{\sum_{p\in Hb}{A}_{i,p}}{{A}_{i,Total}}$, where Hb = {Hba, Hbb1, Hbb2}. PF4 (%Total) was calculated as $100\times \frac{{A}_{i,PF4}}{{A}_{i,Total}}$, and values are displayed as × 10^–3^%Total for readability. The coagulation score (%Total) was calculated as $100\times \frac{\sum_{p\in Coag}{A}_{i,p}}{{A}_{i,Total}}$, where *Coag* = {Fga, Fgb, Fgg, F2}. Plasma proxy (%Total) was calculated as $100\times \frac{\sum_{p\in Plasma}{A}_{i,p}}{{A}_{i,Total}}$, where *Plasma* = {Alb, Fga, Fgb, Fgg, F2, C3, C4, C9}. Coagulation/Hemoglobin is the ratio of the coagulation score to the hemoglobin fraction (equivalent to $\frac{{Coag}_{sum,i}}{{Hb}_{sum,i}}$). Values are mean ± SD (n = 8 per group). The full list of proteins (gene symbols and UniProt accessions) used to compute each index is provided in Supplemental Table 4.

### Network analysis

**Fig. 6:**
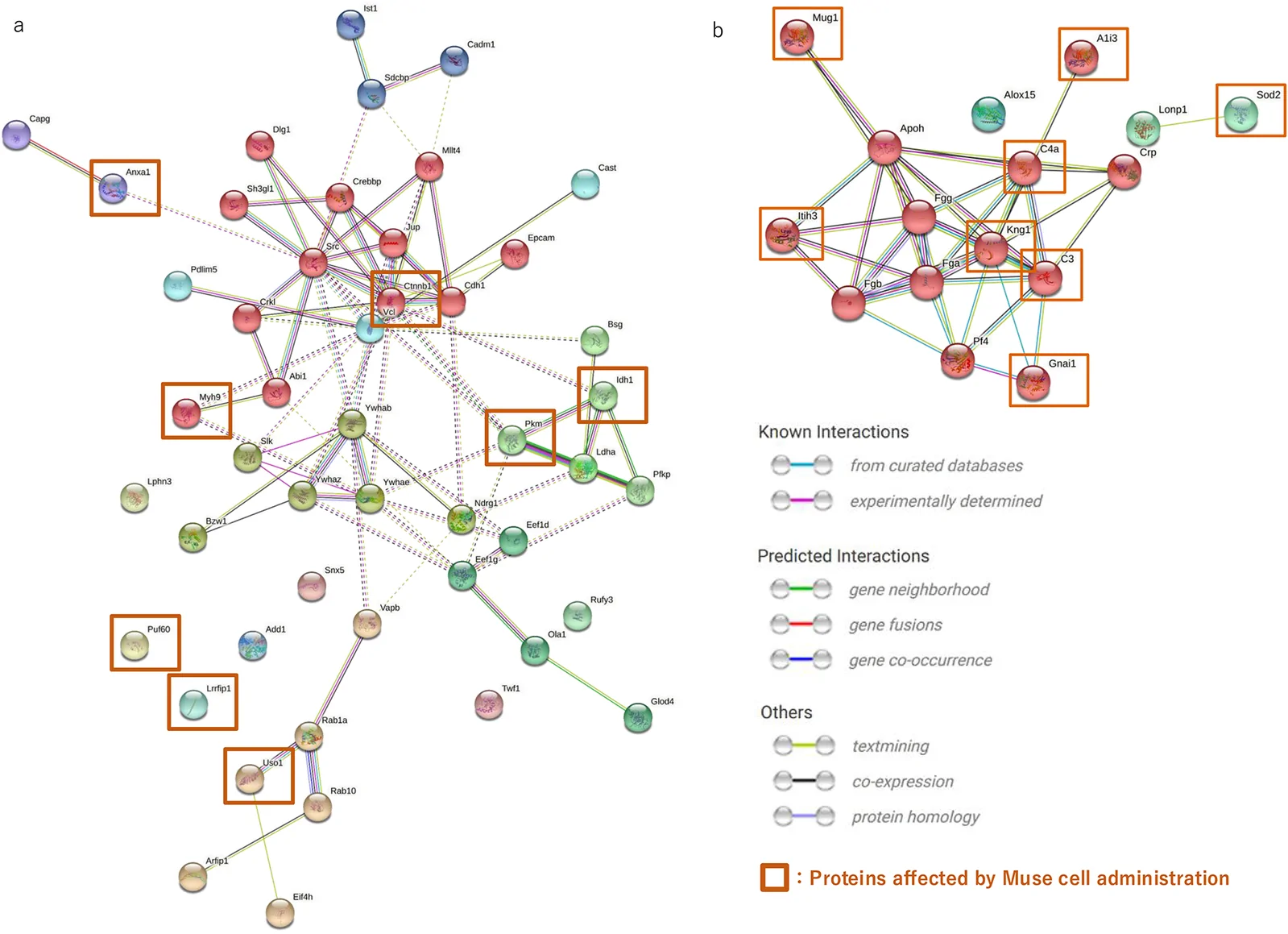
Visualization of molecular networks in the ontologies extracted through the proteomic and functional analyses. **a** The network of proteins included in the cell adhesion-related ontologies. **b** The network of proteins included in the blood coagulation/fibrinolytic system-related ontologies. These networks were mapped and clustered using the Search Tool for the Retrieval of Interacting Genes/Proteins 11.0 (STRING 11.0, https://string-db.org/) with Markov cluster algorithm. Each node represents a single protein, and the length of the edges reflects the closeness of the known or predicted interactions (see the legend for interaction types). Nodes with orange outlines represent the proteins affected by Muse cell administration. Nodes sharing the same color are grouped based on the stronger inter-node relationships, indicating that they share functions or regulatory pathways

To promote understanding of their functional association, the interaction networks among the proteins found during the functional annotation analysis were visualized using STRING and MCL clustering (Fig. 6a, b). This network displayed the molecular interaction among the cell adhesion- and blood coagulation/fibrinolytic system-related proteins affected by hyperoxia. These proteins were found to be closely related to each other. The nodes indicate the individual proteins, and the distance between them indicates the depth of the relationship between the proteins (the shorter the distance, the closer the relationship). The nodes of the same color have a strong relationship, indicating that they are classified in the same group.

Figure 6a illustrates the division of a network of 49 cell adhesion-related proteins affected by hyperoxia (Table 1 cluster 1). The eight proteins (Table 2 cluster 5 and Supplemental Table 1) affected by Muse cell administration were not confined to any particular group, but they were scattered on each group. In the STRING network of the 49 cell adhesion-related proteins (Fig. 6a), the eight Muse-responsive proteins (Table 2, Cluster 5; Supplemental Table 1) were distributed across multiple MCL clusters rather than being confined to a single cluster. Conversely, Fig. 6b also demonstrates that the 17 blood coagulation/fibrinolytic system-related proteins affected by hyperoxia (Table 1 cluster 4) are closely related.

### Histological analysis of Ctnnb1 and Anxa1

**Fig. 7:**
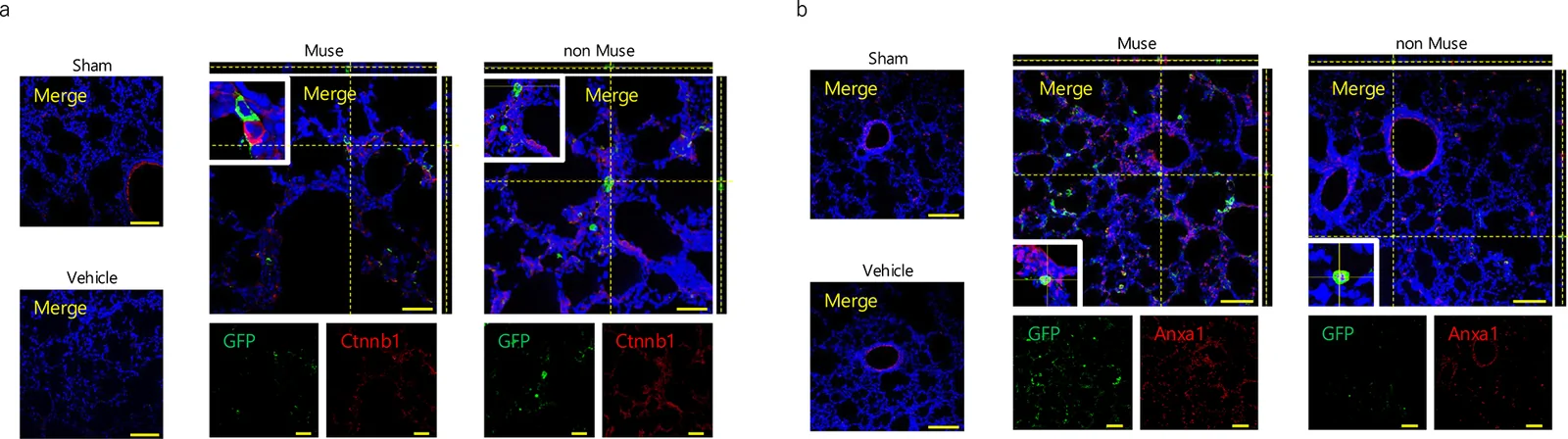
Immunofluorescence staining. Immunofluorescence staining for GFP or Ctnnb1. **a** Histological analysis demonstrated a marked upregulation of Ctnnb1 expression in the cells adjacent to the Muse cells. **b** Immunofluorescence staining for GFP or Anxa1. Histological analysis showed a significant increase in the number of Anxa1-positive cells in the Muse group

A network analysis of cell adhesion-related proteins was performed on the molecules selected from the statistical and annotation analyses of the protein profile. The results revealed that Ctnnb1 (β-catenin) was the most central molecule in the network (Fig. 6a). The expression of this molecule decreased in hyperoxia cases, and this decrease was ameliorated by the stem cell administration (Muse/non-Muse cells) (Fig. 5a). The results of the histological analysis further demonstrated a marked upregulation of Ctnnb1 expression in cells adjacent to the Muse cells (Fig. 7a). Contrarily, although an increase in Ctnnb1 was observed in the non-Muse group, this upregulation was detected at locations distant from the non-Muse cells (Fig. 7a).

Additionally, based on the results of the BALF (Fig. 2d) and proteomic analyses (Supplemental Table 1), we focused on Anxa1 (Annexin A1), a molecule associated with neutrophil migration and inflammatory responses. Anxa1 abundance was reduced in the Vehicle group compared with Sham and was higher in the Muse group compared with Vehicle (Fig. 5a). The results of the histological analysis showed a significant increase in the number of Anxa1-positive cells in the Muse group (Fig. 7b). Although an increase in Anxa1-positive cells was also observed in the non-Muse group, the increase was only minimal, compared to that observed in the Muse group (Fig. 7b). Additionally, Anxa1 expression was observed in a subset of spherical GFP-positive Muse cells.

## Discussion

Although intravenous and intratracheal administrations represent distinct delivery routes and, therefore, strictly different models, precluding a direct comparison, the present study demonstrated comparable improvements in respiratory function, lung histology, and inflammation with Muse cell treatment via both routes. However, intravenous administration was superior to intratracheal administration with respect to survival and body weight gain. Intravenously administered human Muse cells, not non-Muse cells, were engrafted and expressed alveolar epithelial cell markers in the hyperoxia-induced lung injury rat model. The Muse cells ameliorated alveolar growth impairment induced by hyperoxia and improved the pulmonary function. The administration of Muse cells, not the non-Muse cells, suppressed the inflammation caused by hyperoxia. Animals receiving Muse cells intravenously displayed improvement in hyperoxia-associated pulmonary hypertension, as compared to the non-Muse and vehicle groups. A comprehensive analysis of proteins from hyperoxia lungs revealed that Muse cells may have additional effects on the abnormalities in cell adhesion and the blood coagulation/fibrinolytic system caused by hyperoxia. These results demonstrated the histological and functional benefits of human Muse cells in experimental hyperoxia-induced lung injury model.

A major finding of the present study is that the xenogeneic Muse cells were engrafted in the injured lung without the use of immunosuppressants and expressed alveolar epithelial cell markers (podoplanin and PE10). Given that the lungs are the first organs to trap cells following intravenous administration, a human-specific genome material was detected to some extent at 2 weeks, even in our non-Muse group, which differed from previous reports [9, 24–28]. However, the number of cells detected in the lung by the GFP-positive cells at 2 weeks was much higher in the Muse group than in the non-Muse group. The S1P–S1P receptor 2 axis plays an important role in the selective homing of intravenously administered Muse cells into the damaged sites [27, 49]. The S1P expression is increased at the site of injury [24] and in the peripheral blood [50]. The ability of intravenously administered Muse cells to migrate to the injury site is a major advantage over many other types of stem cells. In AluPCR, the Muse cells were detected in large numbers in the lungs, brain, and liver. We believe that this is because the damage caused by high oxygen exposure also occurs in the lungs, brain, and liver, causing Muse cells to migrate to the injured sites. We also believe that the Muse and non-Muse cells are detected in the spleen due to the organ’s filtering function.

Although intravenous and intratracheal administration of Muse cells resulted in largely comparable improvements in respiratory function, lung histology, and inflammatory parameters, intratracheal administration was associated with inferior outcomes in terms of survival and postnatal weight gain. These findings suggest that the experimental intratracheal procedure itself may have influenced the observed outcomes.

In neonatal animal models, intratracheal administration is technically demanding and may impose additional procedural stress and transient respiratory instability, particularly in fragile pups. Thus, the inferior survival and growth observed after intratracheal delivery may reflect, at least in part, limitations related to the administration technique, dosing, or timing rather than a lack of therapeutic efficacy of Muse cells. From a translational perspective, intratracheal administration may also be invasive and stressful in clinical settings, especially in severely ill infants with compromised respiratory status. Therefore, when therapeutic effects are comparable between delivery routes, intravenous administration may represent a safer and more practical option for clinical application. Nevertheless, further studies are warranted to optimize intratracheal delivery strategies, including dose, timing, and procedural techniques, to better define the therapeutic potential and limitations of this route.

In the present study, pulmonary inflammation was assessed using total WBC counts and differential cell counts in BAL fluid, lung histology and immunofluorescence, and cytokine measurement. Macrophages in BALF were increased in the vehicle group and reduced by Muse cell administration, whereas lymphocyte and neutrophil counts did not differ among groups, suggesting a predominant role of macrophages in this model. Consistently, ED1⁺ iNOS⁺ macrophages in lung tissue and serum TNF-α levels were elevated in the vehicle group and attenuated by Muse cells. Together, these findings provide an evaluation of pulmonary inflammation and support the anti-inflammatory effects of Muse cells, potentially through modulation of macrophage-associated responses.

Regarding differentiation, Muse cell differentiates into tissue-compatible cells after homing in various injury models, including stroke [51], neonatal hypoxic–ischemic encephalopathy [9], liver fibrosis [27], acute myocardial infarction [24], chronic kidney disease [26], and aortic aneurysms [52]. Notably, engrafted Muse-derived cells more frequently displayed a flattened/squamous morphology, whereas non‑Muse cells tended to remain spherical in lung sections. This morphological difference may reflect differential interactions with the alveolar niche (e.g., adhesion, spreading, and/or early acquisition of epithelial-like features) and is consistent with the higher retention/engraftment observed in the Muse group. However, cell morphology alone is not sufficient to infer lineage commitment or functional integration, and additional lineage markers and time-course analyses will be required. Regarding hyperoxia-induced lung injury model, the migration of Muse cells into the lungs and their differentiation into alveolar epithelial type 1 and 2 cells have been reported [53]. In the present study, Muse cells also expressed markers for alveolar epithelial type 1 and 2 cells. The Muse cells, such as the MSCs, are also known to secrete various cytokines, producing paracrine effects [9, 24, 26, 54, 55]. Although there are many reports on BPD treated with MSCs, which have demonstrated some treatment effects [16], non-Muse cells did not show sufficient efficacy in the present study. The direct effect of Muse cells to engraft and differentiate into lung cells, in addition to their paracrine effect, may have resulted in a significant therapeutic effect.

In addition, the ring-like distribution of hPDPN immunoreactivity in a subset of GFP-positive cells is compatible with close apposition to alveolar structures and may contribute to alveolar repair and formation. While detection of human epithelial markers (hPDPN and hPE10) supports acquisition of alveolar epithelial cell-associated proteins [53], marker expression alone does not establish full differentiation or functional contribution to alveolar repair. Future studies incorporating additional epithelial lineage markers and functional readouts would strengthen this interpretation.

To further understand the mechanisms underlying the therapeutic effects of Muse cell administration, we conducted a comprehensive proteomic analysis. The annotation of protein expression profiles highlighted the cell adhesion-related proteins as key molecules involved in this process. The pathology of hyperoxia-induced lung injury model is characterized by alveolar simplification and abnormal pulmonary vascularization due to an interrupted pulmonary and alveolar development [1]. Although the pathogenesis of hyperoxia-induced lung injury model is multifactorial, a common pathway involves inflammatory responses [49]. Inflammation triggers the upregulation of cell adhesion molecules and chemotactic proteins that recruit inflammatory cells to the lungs [50]. The interactions between the adhesion molecules on the surfaces of the leukocytes and endothelial cells facilitate the migration of leukocytes from the bloodstream into the lung interstitium, mediating the inflammatory response [38]. Given the evidence for subclinical pulmonary endothelial dysfunction in hyperoxia-induced lung injury model, it may be crucial to evaluate the lung adhesion molecules—an early marker of endothelial dysfunction—in clinical specimens, such as BALF from neonates with established hyperoxia-induced lung injury model. These adhesion molecules, which adhere to the endothelial surface, play a key role in leukocyte infiltration through the endothelial cells and into the lung parenchyma [49].

Among these adhesion-related proteins, network analysis identified Ctnnb1 as the most central node (Fig. 6a). Ctnnb1 is a pivotal molecule in the Wnt/β-catenin signalling pathway, which regulates stem cell pluripotency, differentiation, proliferation, and migration during development [56]. In the respiratory system, Ctnnb1 promotes cellular maturation, differentiation, and morphogenesis [57]. Our findings revealed a significant reduction in Ctnnb1 expression associated with hyperoxia, which was restored following the administration of Muse cells (Figs. 5a and 7). The results of our histological analysis further demonstrated that β-catenin expression was markedly elevated in the alveolar epithelial cells adjacent to the Muse cells that had migrated into the lung tissues (Fig. 7). The spatial pattern of β‑catenin immunoreactivity differed between treatments: enhanced staining was predominantly observed in cells adjacent to Muse cells, whereas the non‑Muse group showed increased β‑catenin at locations more distant from non‑Muse cells. This contrast is consistent with a localized niche effect associated with Muse cell homing and retention (e.g., short-range paracrine signaling and/or direct cell–cell contact) [49], although causal inference is limited by the current cross-sectional design. These results suggest that Muse cell administration may improve lung function by directly or indirectly inducing Wnt/β-catenin signalling in the neighbouring lung cells, either through direct cell interactions or via the paracrine factors.

In addition, inflammatory modulators within the adhesion-related network may also contribute to the observed therapeutic effects. In light of the significant reduction in total leukocyte counts in BALF following Muse cell administration (Figs. 1g and 2d), we focused on Anxa1, a molecule known for its role in regulating inflammation and neutrophil infiltration. This observation prompted us to examine whether Anxa1 upregulation might be associated with the attenuation of pulmonary inflammatory cell infiltration in the Muse group. According to the Human Protein Atlas database (https://www.proteinatlas.org/ENSG00000135046-ANXA1), Anxa1 is strongly expressed in alveolar macrophages and bronchial epithelial cells. Additionally, Anxa1 suppresses immune cell infiltration and promotes the release of the anti-inflammatory cytokine IL-10, which helps reduce pulmonary inflammation [58]. In the present study, we observed a marked upregulation of the Anxa1 expression in some Muse cells that had migrated into the lung (Fig. 7b). This finding suggests that Muse cell administration increases the number of Anxa1-positive cells, potentially limiting excessive immune cell infiltration and the accompanying inflammation. This mechanism may underlie the greater improvement in lung function and development in comparison to the non-Muse group. These findings align with the reduction in leukocyte counts observed in the BALF (Fig. 2d), supporting a potential role of Anxa1 in the beneficial effects of Muse cells in hyperoxia. Because ANXA1 immunoreactivity was detected in a subset of GFP-positive Muse cells, the increased number of ANXA1-positive cells may reflect both donor-cell expression and induction of ANXA1 in neighboring host cells; distinguishing these possibilities (e.g., species-specific probes or co-staining with host-cell markers) will be important in future studies.

Beyond the cell adhesion-related proteins, the blood coagulation and fibrinolytic system-related proteins are also important to understand the effects of Muse cell administration in hyperoxia. Among the 17 proteins analyzed, nine were significantly impacted by the Muse cell treatment (Table 2, Cluster 3; Supplemental Table 2), whereas eight were unaffected (Supplemental Table 3). The unaffected proteins, which all promote blood coagulation, were upregulated in response to hyperoxia (Fig. 5c). Conversely, four out of the nine proteins influenced by Muse cell administration—Kng1, Gnai3, Map1, and Sod2—inhibit coagulation and promote vasodilation, and their expression was further upregulated following Muse cell treatment (Fig. 5b). These proteomic changes are compatible with a Muse cell-associated shift in the balance of proteins implicated in the regulation of coagulation/fibrinolysis and vasoreactivity; however, no functional coagulation or fibrinolysis assays were performed, and therefore functional effects on hemostasis or pulmonary microcirculatory flow cannot be concluded from the present dataset. A molecular network analysis (Fig. 6b) indicated that most of the 17 proteins are closely interrelated, implying that Muse cells may induce compensatory changes in an alternate group of blood coagulation and fibrinolytic proteins. Indeed, the suppression of plasminogen activator-mediated fibrinolysis not only increases the fibrin deposition in the alveoli but also contributes to the recruitment and migration of inflammatory cells [59]. Fibrinolytic therapies aimed at preventing pulmonary and pleural fibrin deposition, along with inflammation, show considerable promise [60, 61].

Collectively, our GO-term enrichment analysis showed that adhesion-related and blood coagulation/fibrinolysis-related ontologies were enriched among hyperoxia-affected proteins (Group I) and were also recovered among hyperoxia-affected proteins that were responsive to Muse treatment (Group III), whereas these ontologies were not among the enriched clusters for the corresponding non-Muse–responsive proteins (Group V + VII). This pattern is consistent with a Muse-associated modulation of protein sets linked to cell–cell/cell–matrix interactions and coagulation/fibrinolysis-related vascular-inflammatory processes in the injured lung. In contrast, filament- and cytoskeleton-related ontologies were enriched in both Muse- and non-Muse–responsive groups, suggesting that structural remodeling represents a broader MSC-associated response. Together, these findings support a model in which Muse cells exert distinct regulatory effects on adhesion and vascular-inflammatory pathways, beyond the shared cytoskeletal remodeling induced by MSCs in general. However, because GO-term enrichment derived from bulk tissue proteomics lacks cellular resolution and does not establish function, targeted validation and cell-type–specific localization will be required to define the cellular sources and functional relevance of these pathway shifts.

In addition, our data do not support variable erythrocyte or platelet carryover as the primary driver of the coagulation/fibrinolysis-related signal (Supplemental Table 4). Erythrocyte- and platelet-enriched markers (EMI and PF4) were not increased in the hyperoxia-exposed groups (Table 4). Moreover, across all samples, the summed raw abundance of hemoglobin subunits (*Hb*_*sum*_) showed no evidence of an association with the summed raw abundance of core coagulation proteins (*Coag*_*sum*_) (Spearman’s rs = 0.014, p = 0.94; n = 32). These analyses indicate that the observed coagulation/fibrinolysis-related signal is unlikely to be a technical artefact driven by variable residual intravascular blood carryover. Therefore, the observed enrichment of coagulation/fibrinolysis-related proteins is more consistent with disease-related changes—such as increased vascular permeability and/or local activation of intrapulmonary coagulation pathways—than with sample-to-sample differences in residual blood content. We note that vascular perfusion can reduce intravascular blood-derived proteins, but it may also alter tissue proteome profiles in a tissue-dependent manner; therefore, we explicitly evaluated blood-derived signatures within our dataset.

This study has several limitations. First, this study is limited by the use of a hyperoxia-induced neonatal lung injury model, which does not fully replicate the clinical complexity of bronchopulmonary dysplasia (BPD). However, this model is widely used and accepted as an experimental surrogate of BPD, as it reproduces several key pathological features relevant to BPD pathophysiology. Previous studies have demonstrated that this model exhibits impaired alveolar development, characterized by reduced alveolar number and enlarged alveolar spaces, as well as disrupted pulmonary vascular growth, pulmonary hypertension, increased pulmonary vascular permeability, and inflammatory cell infiltration [16]. Owing to these well-established structural and vascular abnormalities, the model remains a useful platform for evaluating therapeutic strategies, including the effects of Muse cells in the present study.

Second, this study is limited by the intratracheal administration procedure. Although intravenous and intratracheal delivery of Muse cells produced largely comparable improvements in respiratory, histological, and inflammatory outcomes, inferior survival and postnatal weight gain were observed after intratracheal administration. These findings may, at least in part, reflect procedure-related factors inherent to intratracheal delivery in neonatal animals, including technical difficulty, procedural stress, and transient respiratory instability, rather than reduced therapeutic efficacy of Muse cells. Accordingly, the interpretation of the intratracheal data should be made with caution.

## Conclusion

Intravenous administration of Muse cells demonstrated superior therapeutic effects in a hyperoxia-induced lung injury model compared with non-Muse cells. Muse cells ameliorated respiratory impairment and pulmonary hypertension, accompanied by differentiation into alveolar epithelial cells and modulation of cell adhesion and coagulation pathways. Our study findings suggest the potential of Muse cell therapy as a promising approach for treating severe neonatal lung diseases and warrant further translational and clinical investigations to validate the results.

## Supplementary Information

Supplementary Material 1.

Supplementary Material 2.

Supplementary Material 3.

Supplementary Material 4.

Supplementary Material 5.

Supplementary Material 6.

## Data Availability

All data are available from the corresponding author upon reasonable request. Raw proteomic data were submitted to the Japan Proteome Standard Repository/Database (https://repository.jpostdb.org/). The accession number of jPOST for the proteomic data used in the present study is JPST003674 (PXD061493).

## Abbreviations

BPD: Bronchopulmonary Dysplasia
Muse cell: Multilineage-differentiating stress enduring cell
MSC: Mesenchymal Stromal Cell
SSEA: Stage-Specific Embryonic Antigen
VLBW: Very Low Birth Weight
S1P: Sphingosine-1-Phosphate
HLA: Human Leukocyte Antigen
BM-MSC: Bone Marrow- Mesenchymal Stromal Cell
GFP: Green Fluorescent Protein
FACS: Fluorescence-Activated Cell Sorter
BALF: Bronchoalveolar Lavage Fluid
TV: Tidal Volume
WBP: Whole Body Plethysmography
TVD: Tissue Volume Density
qPCR: Quantitative Polymerase Chain Reaction
PBS: Phosphate-Buffered Saline
RV: Right Ventricle
LV: Left Ventricle
IVS: Interventricular Septum
RVSP: Right Ventricular Systolic Pressure
LC/MS/MS: Liquid Chromatography/Tandem Mass Spectrometry
GO: Gene Ontology
iv: Intravenous
it: Intratracheal
