# Supplementary material for: Intravenously delivered multilineage-differentiating stress-enduring cells dampen hyperoxia-induced lung injury in a rat model with features of bronchopulmonary dysplasia
Source: Stem Cell Res Ther. 2026 Sep 4;17:300. doi: 10.1186/s13287-026-05086-2 (PMC13545779; doi:10.1186/s13287-026-05086-2)
Supplement: Supplementary file 1 [file 13287_2026_5086_MOESM1_ESM.pdf]

Lonza Walkersville, Inc.
www.lonza.com
scientific.support@lonza.com
Scientific Support: 800-521-0390
Document # PPT 101-10 08/10
Walkersville, MD 21793-0127 USA
© 2011 Lonza Walkersville, Inc.

Poietics™ Human Bone Marrow

THESE PRODUCTS ARE FOR RESEARCH USE ONLY. Not approved for human or veterinary use, for application to humans or animals, or for use in clinical or *in vitro* procedures.

Lonza Walkersville, Inc. operates a Research Bone Marrow Donor Program to recruit, screen, test, evaluate and select donors for collection of bone marrow samples **for research use**. Donors participate in a two stage process: 1) evaluation at a screening appointment, 2) marrow collection.

Donation informed consent to participate in the bone marrow donor program includes:

- Program explanation with questions and answers about the process
- Health assessment through medical and behavioral questionnaire
- Permission for blood sample collection and testing by certain infectious disease tests
- Confidentiality of identity and protection of health information
- Risks and discomforts of participation
- Benefits of participation do not include any direct benefits or ownership
- Donor may stop participation in the program at any time
- Donor's options for continued program participation
- Reasonable compensation for participation
- Bone marrow will be sold to laboratories performing research

This Donor Program is currently approved, has been approved for over 10 years, and is submitted for annual approval by a commercial institutional review board.

Lonza Walkersville, Inc. 8830 Biggs Ford Road, Walkersville, MD 21793 is licensed as a tissue bank to conduct this donor program in the states of Maryland and New York, US.

Please address questions/comments on this information to Lonza Scientific Support.

Ficoll-Paque® is a registered trademark of GE Healthcare BIO-Sciences AB. MyeloCult is a registered trademark of StemCell Technologies. All other trademarks herein are marks of Lonza Group or its subsidiaries.

Source

Human bone marrow withdrawn from bilateral punctures of the posterior iliac crests of normal volunteers.

Normal Donor Eligibility Criteria

- Healthy males and non-pregnant females between the ages of 18 and 45 years old
- Signed and witnessed informed consent forms
- Acceptable vital signs and hematology values
- All donors are screened for general health and negative medical history for heart disease, kidney disease, liver disease, cancer, epilepsy and blood or bleeding disorders
- Negative clinical laboratory tests for HIV-1, HIV-2, hepatitis B and hepatitis C

Bone Marrow Sample Collection

Bone marrow is collected into syringes containing heparin (~100 units per ml bone marrow).

Scheduling

Fresh cells need to be ordered two to four weeks in advance depending on donor and volume requirements.

Products

Available as unprocessed marrow, mononuclear cells, isolated hematopoietic progenitor cells ($CD34^+$, $CD133^+$), mesenchymal stem cells and stromal cells.

Product Uses

Researchers may use these for:

- hematopoietic growth factor studies
- clonogenic assays
- long-term culture initiating-cell assays (LTC-IC)
- progenitor cell proliferation
- progenitor cell differentiation
- stem cell expansion
- hematopoietic microenvironment studies
- gene therapy research
- drug screening
- lead optimization
- high-throughput screening
- toxicology

Product Preparation / Description

Unprocessed marrow

Human Unprocessed Bone Marrow is supplied in tubes and shipped fresh overnight. Also available in gas permeable bags.

- o 10 ml (1M-105)
- o 25 ml (1M-125)
- o 4 x 25 ml same donor (4x1M-125)

Mononuclear cells

Fresh Bone Marrow Mononuclear Cells

For Bone Marrow Mononuclear Cells, bone marrow is diluted to 5-10 million nucleated cells per ml in HBSS. Cells are layered over Ficoll-Paque® and centrifuged. The mononuclear cell layer is removed and washed in HBSS. Cells are resuspended in HBSS containing 0.5% BSA and 5 mM EDTA, and shipped at 4 to 8°C the same day the marrow is collected for next day delivery. Available in the following sizes:

- o ≥ 25 million (1M-125C)
- o ≥ 100 million (1M-125D)
- o ≥ 200 million (1M-125A)
- o ≥ 300 million (1M-125E)

Cryopreserved Bone Marrow Mononuclear Cells

Cryopreserved Bone Marrow Mononuclear Cells are available cryopreserved in the following sizes:

- o ≥ 5 million cells (2S-101D)
- o ≥ 25 million cells (2M-125C)
- o ≥ 100 million cells (2M-125D)
- o ≥ 200 million cells (2M-125A)
- o ≥ 300 million cells (2M-125E)
- o There is also a "sampler pack" of 5 donors, ≥ 25 million cells each in 5 separate vials (2M-125B).

Cryopreserved Mesenchymal Stem Cells

Bone marrow contains a population of rare progenitor cells known as Mesenchymal Stem Cells (MSC) capable of replication as undifferentiated cells or differentiating into bone, cartilage, fat, muscle, tendon and marrow stroma. Available in the following size:

- o ≥ 750,000 cells (PT-2501)

$CD34^+$ cells

$CD34^+$ progenitor cells are isolated from bone marrow mononuclear cells using positive immunomagnetic selection. Purity is ≥ 95%, and the cells are cryopreserved in the following sizes:

- o ≥ 100,000 cells (2M-101)
- o ≥ 300,000 cells (2M-101A)
- o ≥ 500,000 cells (2M-101B)
- o ≥ 1 million cells (2M-101C)
- o ≥ 2 million cells (2M-101D)

$CD133^+$ cells

$CD133^+$ progenitor cells are isolated from bone marrow mononuclear cells using positive immunomagnetic selection. Purity is ≥ 90%, and the cells are cryopreserved in the following sizes:

- o ≥ 100,000 cells (2M-102A)
- o ≥ 500,000 cells (2M-102)

Stromal cells

These stromal cells function as a feeder layer for hematopoietic progenitors, allowing proliferation and differentiation of progenitors to continue for weeks in these cultures with no addition of exogenous cytokines. Fresh mononuclear cells are put into liquid culture for 3 to 4 weeks at $2x10^6$ cells/ml using StemCell Technologies' MyeloCult® long term culture medium. The cells are harvested and cryopreserved. Available in the following size:

- o ≥ 5 million cells (2M-302)

Product Warranty

CULTURES HAVE A FINITE LIFESPAN *IN VITRO*. Lonza warrants its cells only if Poietics™ Medium is used, and when recommended protocols are followed. Cryopreserved human bone marrow cells are assured to be viable and functional when thawed and maintained properly.

THESE PRODUCTS ARE FOR RESEARCH USE ONLY. Not approved for human or veterinary use, for application to humans or animals, or for use in clinical or *in vitro* procedures.

WARNING: POIETICS™ HUMAN BONE MARROW PRODUCTS CONTAIN HUMAN SOURCE MATERIAL, TREAT AS POTENTIALLY INFECTIOUS. Each donor is tested and found non-reactive for the presence of HIV-I, HIV-2, Hepatitis B Virus and Hepatitis C Virus performed in a CLIA laboratory. Testing can not offer complete assurance that HIV, Hepatitis B, and Hepatitis C Viruses are absent. All human sourced products should be handled at the Biological Safety Level 2 to minimize exposure of potentially infectious products, as recommended in the CDC-NIH Manual, Biosafety in Microbiological and Biomedical Laboratories, 5th ed. If you require further information, please contact your site Safety Officer or Scientific Support.

Ficoll-Paque® is a registered trademark of GE Healthcare BIO-Sciences AB. MyeloCult is a registered trademark of StemCell Technologies. All other trademarks herein are marks of Lonza Group or its subsidiaries.
