# Supplementary material for: Intravenously delivered multilineage-differentiating stress-enduring cells dampen hyperoxia-induced lung injury in a rat model with features of bronchopulmonary dysplasia
Source: Stem Cell Res Ther. 2026 Sep 4;17:300. doi: 10.1186/s13287-026-05086-2 (PMC13545779; doi:10.1186/s13287-026-05086-2)
Supplement: Supplementary file 2 [file 13287_2026_5086_MOESM2_ESM.pdf]

Lonza Walkersville, Inc.
8830 Biggs Ford Road
Walkersville, MD 21793
Tel +1 301 898 7025

Acquisition of Human Tissue for Research Cell Products

Ethics and Law

- Established ethical practices of the donation and transplantation organizations in the US (AATB*, AOPO*, EBAA*) are followed at Lonza.
- Informed consent, legal authorization and protection of human subjects considerations are followed during all steps of the tissue acquisition process.
- Protected health information is maintained confidentially to protect the privacy of donors as intended by HIPAA* regulation.
- Lonza holds a permit to operate a tissue bank in the state of Maryland and a tissue bank license from the state of New York for non-transplant anatomic tissue.

Tissue Sources

- Human tissue is acquired from tissue recovery agencies, tissue suppliers and Lonza managed donor programs that perform tissue recovery and donor informed consent in accordance with processes approved by an Institutional Review Board or comparable independent review body, where applicable.
- Good business practices are followed to identify, evaluate, qualify, monitor and maintain agreements with tissue sources.
- Each qualified tissue source works with Lonza under specific terms defined in a written agreement that includes donor confidentiality, tissue ownership and use, and informed consent or donation permission.

Permission for Research Use

- A properly executed record of informed consent from living donors, or authorization for donation from the donor or authorizing person for deceased donors, is required for each human tissue received at Lonza.
- Research use is the intended use of this tissue and is documented.
- When a qualified tissue supplier obtains permission for tissue donation either by consent or authorization, that agency maintains the original record and may provide a copy or an attestation statement to Lonza.
- When the Lonza Donor Program obtains informed consent, the original signed record is retained by Lonza.
- Informed consent records contain the required elements of informed consent as stipulated in the US regulations for the protection of human subjects 45 CFR* Part 46 and 21 CFR Parts 50 & 56.
- Authorization records include appropriate language and process as intended in the US Uniform Anatomical Gift Act (UAGA*) (1987) and in updated versions (2006) as enacted by different states.

Donor De-Identification

- Lonza ensures that each donor can be identified to a particular tissue and to all cell products derived from that tissue to fulfill tracking requirements.
- To protect donor identity, tissues and cell products derived from such tissues are coded.
- Access to donor records and coding is restricted.

- Product identification codes can be linked to specific attributes of the donor, such as gender, age, medically relevant information or infectious disease test results to provide that information to the customer.

Non-payment for Human Tissue

- Neither living donors, nor family members, nor estates of deceased donors receive valuable consideration for providing tissue.
- Fees are paid to tissue sources as reimbursement for the medical, technical and transportation services and supplies needed to recover and provide tissue to Lonza.
- For Lonza Donor Programs, no payment is given to donors whose involvement in providing tissue for research is limited to informed consent.
    - Compensation is provided to living donors when additional services are necessary, such as for tissue donor screening, blood sample collection or tissue recovery that may include time and travel.

Certificate of Analysis

Customers receive a Certificate of Analysis (COA) with each cell product derived from human tissue. The COA contains specific technical information and quality control results pertinent to that product. In addition, the following statements are applicable to all of Lonza's research cell products:

> **These cells were isolated from donated human tissue after obtaining permission for their use in research applications by informed consent or legal authorization. This product is for research use only.** Details concerning the use of our cell and media products can be downloaded from our website at www.lonza.com

Research Use Only

For research cell products provided by Lonza, we hold donor consent and legal authorization that provides permission for all research use. The consent and authorization documents for Lonza's research cell products do not identify specific types of research testing that can or cannot be performed. If the customer is using Lonza's cells for research purposes only, this donor consent applies. The researcher is responsible for the testing and type of research performed on the cells.

Non-restricted Use

For Research Use Only indicates that there are no restrictions for the use of Lonza's research cells as long as they are used for research purposes only and not used for diagnostic procedures, drug purposes, commercial purposes, or for administration to humans. Refer to Lonza’s Terms and Conditions of use.

Authorization

To protect privacy of donors, tissue suppliers, and our proprietary processes, Lonza does not provide copies of donor records or tissue source agreements to customers. This document and/or quality audit practices are offered to customers to provide additional assurance of process integrity.

Abbreviations*
AATB: American Association of Tissue Banks
AOPO: Association of Organ Procurement Organizations
EBAA: Eye Bank Association of America
HIPAA: Health Insurance Portability and Accountability Act of 1996
CFR: Code of Federal Regulations
UAGA: Uniform Anatomical Gift Act (1987, revised 2006)
