# Supplementary material for: Intravenously delivered multilineage-differentiating stress-enduring cells dampen hyperoxia-induced lung injury in a rat model with features of bronchopulmonary dysplasia
Source: Stem Cell Res Ther. 2026 Sep 4;17:300. doi: 10.1186/s13287-026-05086-2 (PMC13545779; doi:10.1186/s13287-026-05086-2)
Supplement: Supplementary file 3 [file 13287_2026_5086_MOESM6_ESM.pdf]

A

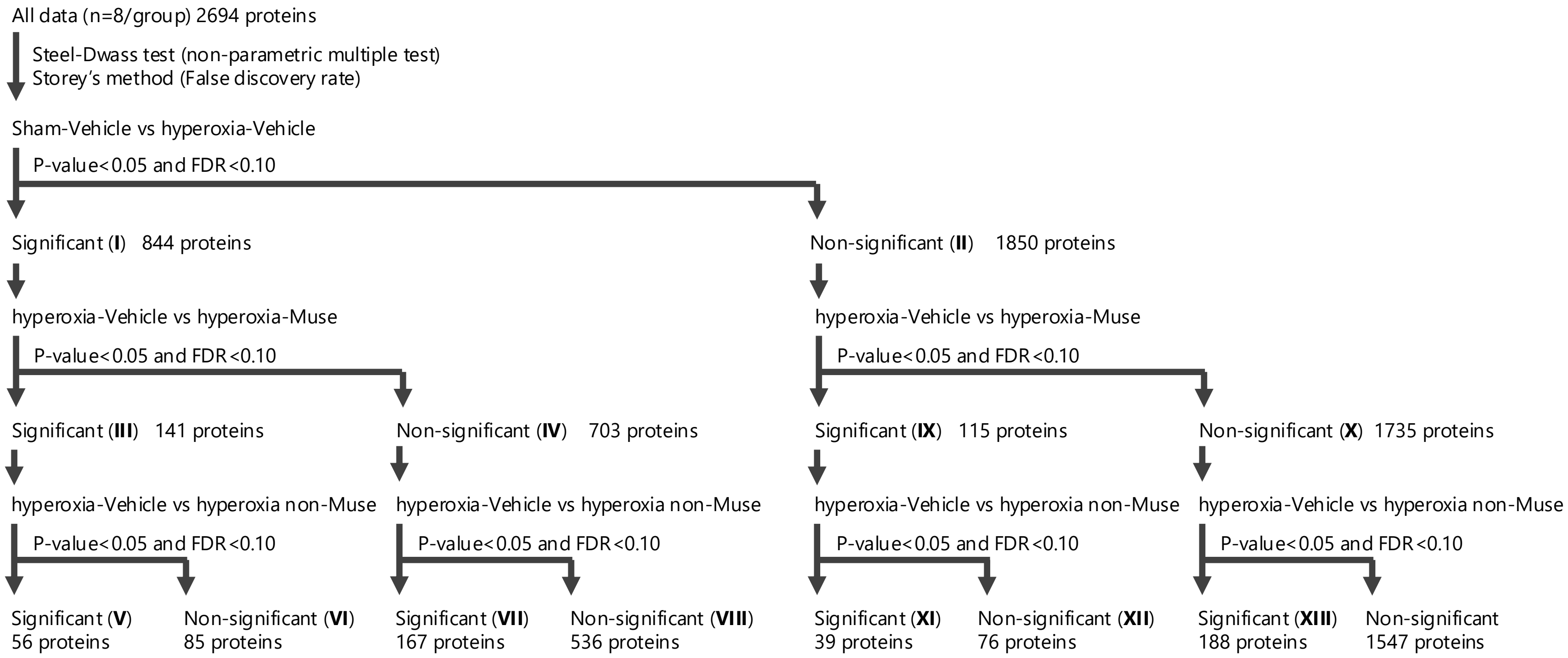

B

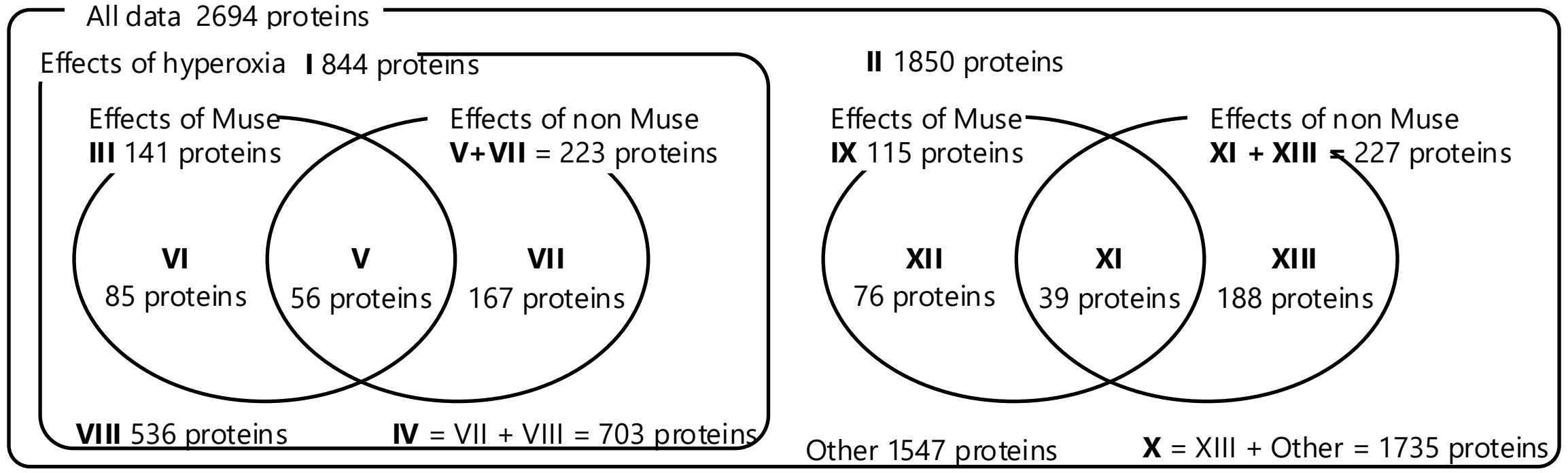

C

	**Proteins affected by hyperoxia 844 proteins**	**Proteins not affected by hyperoxia 1850 proteins**	**Enrichment ratio**
**Proteins affected by Muse 256 proteins**	141 (16.7%)	115 (6.2%)	2.69
**Proteins affected by non-Muse 450 proteins**	223 (26.4%)	227 (12.3%)	2.15

Supplemental Fig1

Supplemental Fig. 1: Stepwise extraction criteria and yield distribution of proteins in proteomic analysis.

(a) A flowchart illustrating the stratified analysis of 2,694 proteins detected by LC-MS/MS. First, based on the comparison between the sham and hyperoxia-vehicle groups ($p < 0.05$, FDR < 0.10), proteins were classified into those significantly affected by hyperoxia (844 proteins) and those not significantly affected by hyperoxia (1,850 proteins). Each subset was then further divided based on whether the proteins were significantly affected by Muse cells (hyperoxia-vehicle vs. hyperoxia-Muse) or by non-Muse MSCs (hyperoxia -vehicle vs. hyperoxia-non-Muse). The resulting categories (I–XIII) cover all combinations of the “affected or unaffected by hyperoxia” and “affected or unaffected by Muse/non-Muse cells.”

(b) A Venn diagram summarizing the classification of proteins in each subset. For example, among the 844 proteins affected by hyperoxia, 141 were significantly affected by Muse cell administration; of which, 56 were also significantly affected by the non-Muse cells. The 1,850 proteins not significantly affected by hyperoxia were classified in the same manner.

(c) A summary table showing the number (and percentage) of proteins in each major classification, along with the calculated enrichment ratio.

Supplemental Fig1

a b

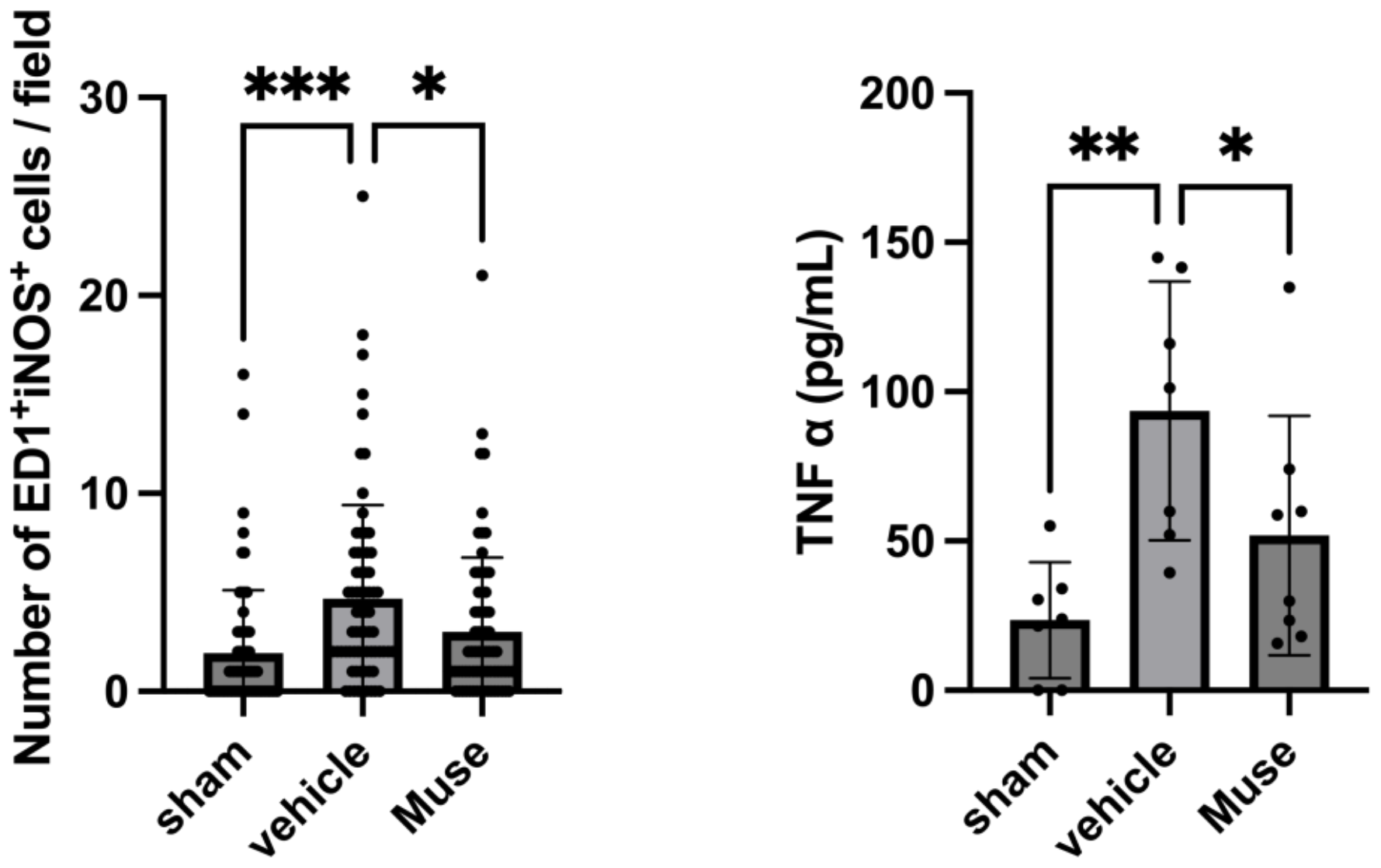

Supplemental Fig. 2:

(a)The number of ED1⁺ iNOS⁺ cell in the hyperoxia-induced lung injury model rats were elevated in the vehicle group compared with the sham group, and this increase was ameliorated by Muse cell administration. (n = 11 for sham, 12 for vehicle and 11 for Muse). *p < 0.05, ***p < 0.001

(b)Serum TNF-α levels in the hyperoxia rats were elevated in the vehicle group compared with the sham group, and this increase was ameliorated by Muse cell administration. (n = 7 for sham, 7 for vehicle and 8 for Muse). *p < 0.05, **p < 0.01

Supplemental Fig2

(a)

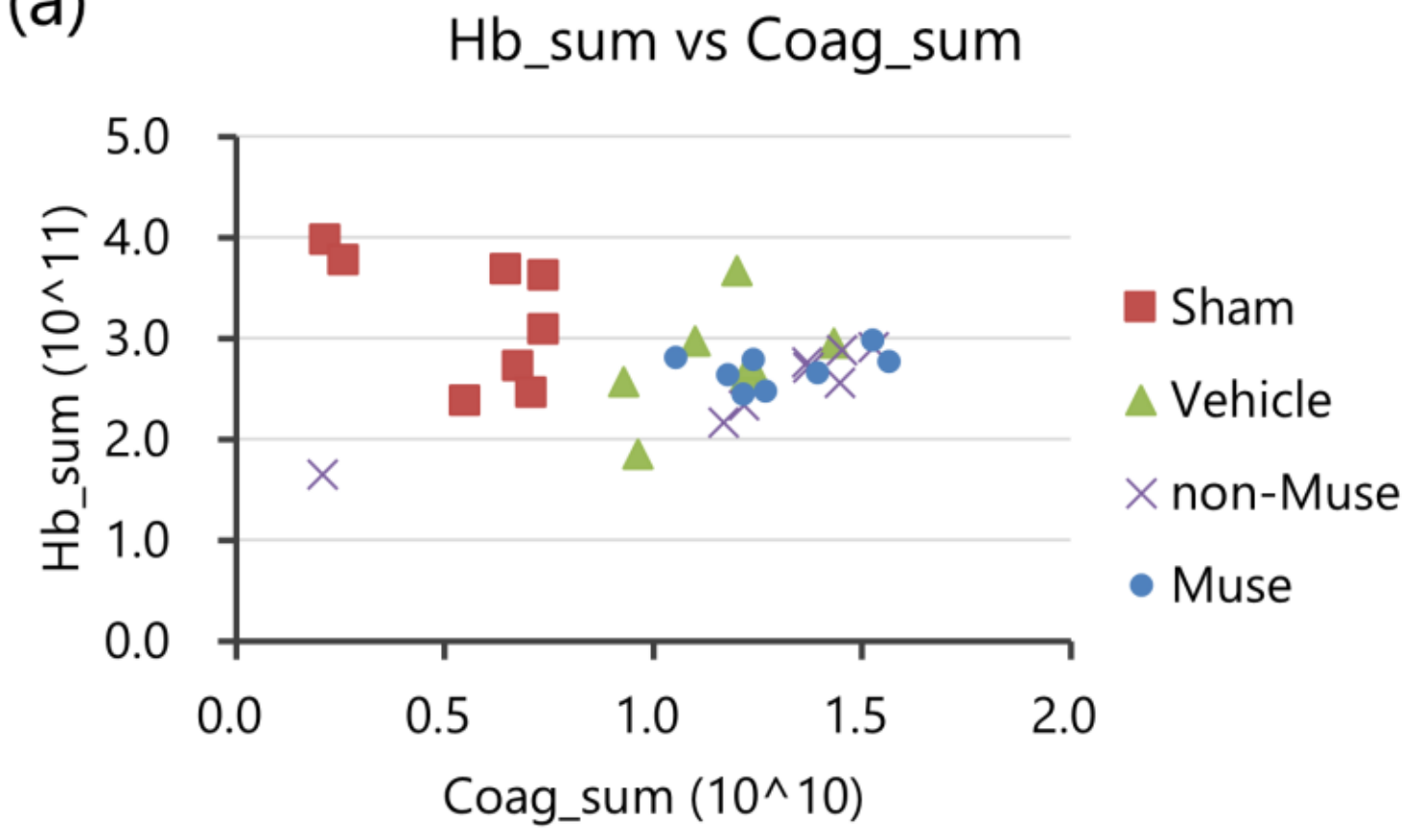

(b)

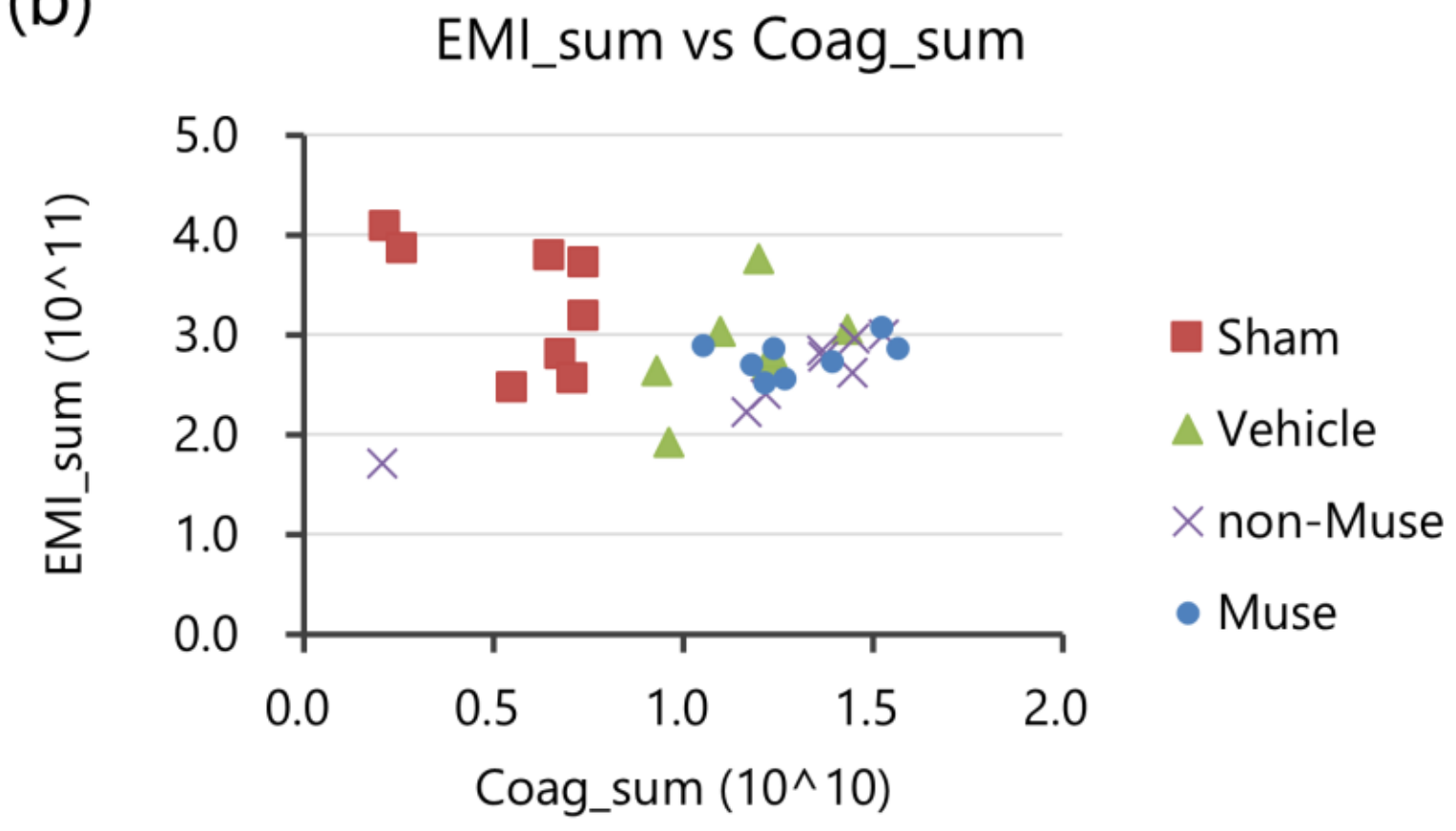

(c)

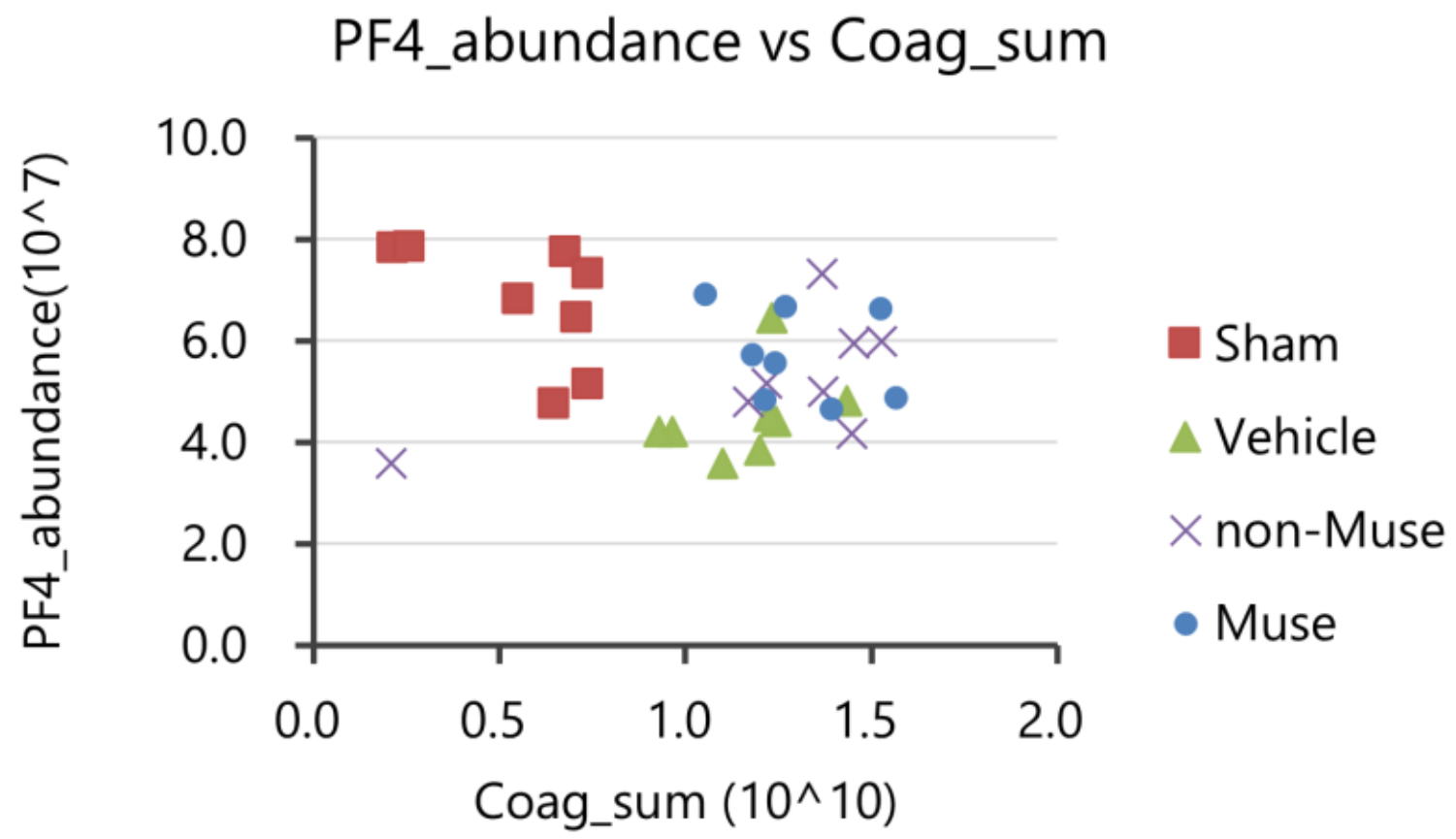

(d)

Comparison	Spearman's rs	p-value
**Hb_sum vs Coag_sum**	0.014	0.941
**EMI_sum vs Coag_sum**	0.018	0.921
**PF4 vs Coag_sum**	−0.137	0.454

Supplemental Fig. 3: Relationships between blood-derived marker abundances and coagulation-related protein abundance in lung tissue proteomics. Scatter plots show
(a) Hb_sum (HBB1 + HBB2 + HBA1/2) vs Coag_sum (FGA + FGB + FGG + F2),
(b) EMI_sum (sum of erythrocyte marker proteins: HBB1, HBB2, HBA1/2, CA1, SLC4A1, GYPC, PRDX2) vs Coag_sum, and
(c) PF4 abundance vs Coag_sum.
(d) Spearman's rank correlation coefficient (rs) and two-sided p value. Each dot represents an individual sample (Sham, Vehicle, non-Muse, and Muse; n=8 per group). Protein abundances reported as not detected (n.d.; below the quantification limit) were imputed with the quantification limit value (55,671.01) prior to calculating summed indices.

Supplemental Fig3

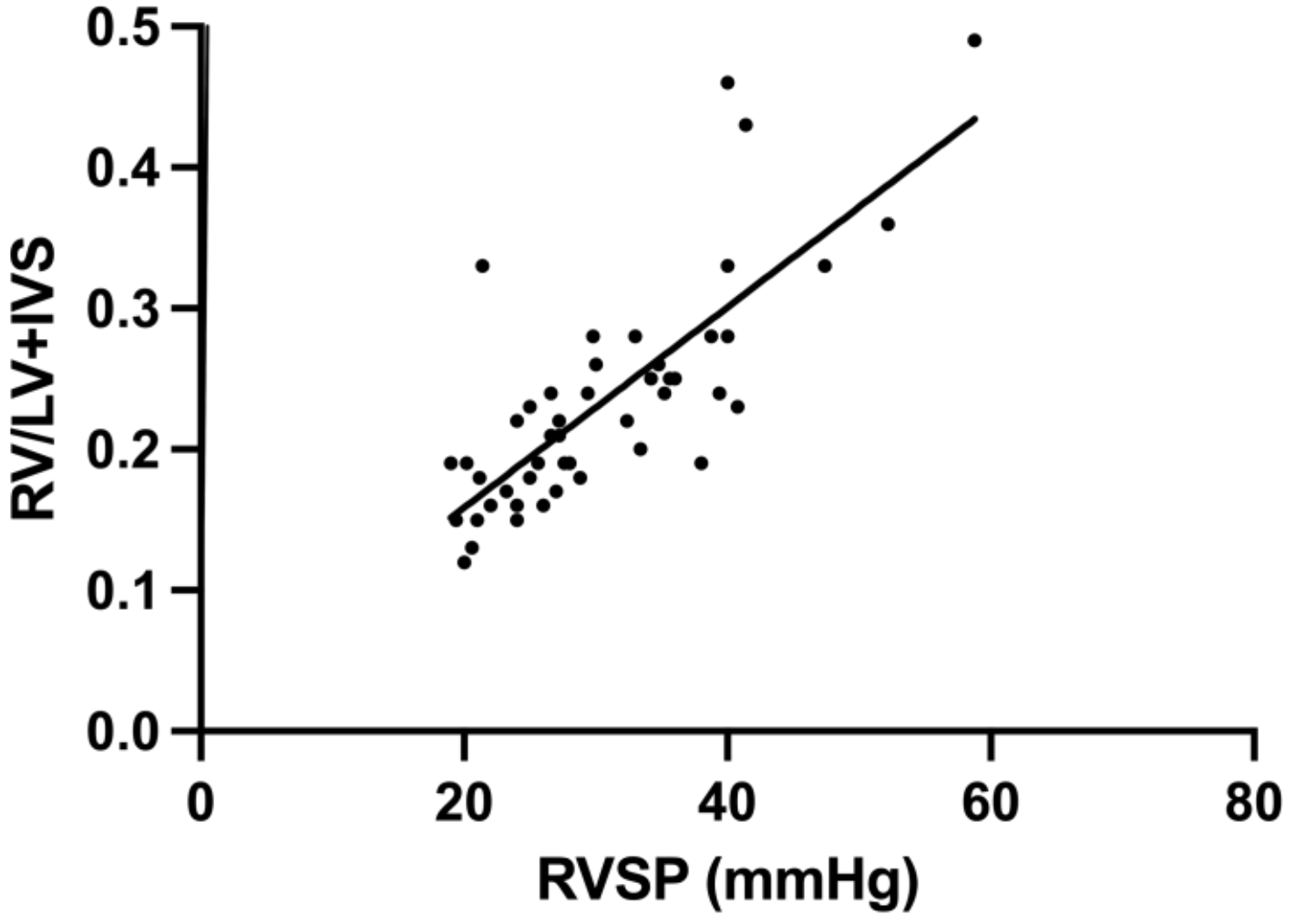

Supplemental Fig. 4: RV/LV+IVS showed a strong correlation with RVSP. ($r = 0.78$, $p < 0.0001$)

Supplemental Fig4
