# Supplementary material for: Intravenously delivered multilineage-differentiating stress-enduring cells dampen hyperoxia-induced lung injury in a rat model with features of bronchopulmonary dysplasia
Source: Stem Cell Res Ther. 2026 Sep 4;17:300. doi: 10.1186/s13287-026-05086-2 (PMC13545779; doi:10.1186/s13287-026-05086-2)
Supplement: Supplementary file 4 [file 13287_2026_5086_MOESM3_ESM.docx]

| Protein | Distribution | | | Main function |
| --- | --- | --- | --- | --- |
| Name | Alveolar cells | Bronchus | Macrophages |  |
| Lrrfip1 | Medium | High | High | Regulation of TNF, EGFR and PDGFA expression and smooth muscle cells proliferation following artery injury through PDGFA repression |
| Myh9 | High | Low | Medium | Cellular myosin that appears to play a role in cytokinesis, cell shape, and specialized functions such as secretion and capping. |
| Uso1 | Low | Medium | Medium | General vesicular transport factor required for intercisternal transport in the Golgi stack |
| Idh1 | Low | Low | Low | Instructions for making an enzyme called isocitrate dehydrogenase 1 |
| Ctnnb1 | High | Medium | Medium | Key downstream component of the canonical Wnt signaling pathway |
| Anxa1 | Medium | High | High | Innate immune response as effector of glucocorticoid-mediated responses and regulator of the inflammatory process. Promotion of chemotaxis of granulocytes and monocytes |
| Puf60 | Medium | Medium | Medium | DNA- and RNA-binding protein, involved in several nuclear processes such as pre-mRNA splicing, apoptosis and transcription regulation. |
| Pkm | Medium | Medium | Medium | Glycolytic enzyme that catalyzes the transfer of a phosphoryl group from phosphoenolpyruvate (PEP) to ADP, generating ATP |

Supplemental table 1. Cell adhesion–related proteins affected by Muse cell injection: distribution and function.

| Protein | Distribution | | | Main function |
| --- | --- | --- | --- | --- |
| Name | Alveolar cells | Bronchus | Macrophages |  |
| Gnai3 | Medium | Medium | Not detected | Transducers downstream of G protein-coupled receptors. |
| Sod2 | Medium | Medium | Medium | Destroys superoxide anion radicals which are normally produced within the cells and which are toxic to biological systems. |
| Map1 | Low | Medium | Medium | Structural protein involved in the filamentous cross-bridging between microtubules and other skeletal elements. Microtubule-associated protein that mediates aggregation of mitochondria resulting in cell death and genomic destruction |
| Kng1 | Not detected | Not detected | Not detected | Inhibitors of thiol proteases. Important role in blood coagulation and inhibition of the thrombin- and plasmin-induced aggregation of thrombocytes. |
| Itih3 | Not detected | Not detected | Not detected | Carrier of hyaluronan in serum or as a binding protein between hyaluronan and other matrix protein. |
| C3 | Not detected | Not detected | Not detected | Central role in the activation of the complement system and the mediation of local inflammatory process. Induction of the contraction of smooth muscle, increases vascular permeability and causes histamine release from mast cells and basophilic leukocytes. |
| C4 | Not detected | Not detected | Not detected | Essential for the propagation of the classical complement pathway. Covalently binds to immunoglobulins and immune complexes and enhances the solubilization of immune aggregates. |
| A1i3 | no data | no data | no data | Protease inhibitor with a wide spectrum of protein targets, which attaches through its thioester function. |
| Mug1 | no data | no data | no data | Protease inhibitor of specific proteolysis in the bait region. |

Supplemental table 2. Blood coagulation and fibrinolytic system–related proteins affected by Muse cell injection: distribution and function.

| Protein | Distribution | | | Main function |
| --- | --- | --- | --- | --- |
| Name | Alveolar cells | Bronchus | Macrophages |  |
| Alox15 | Not detected | High | Low | Non-heme iron-containing dioxygenase that catalyzes the stereo-specific peroxidation of free and esterified polyunsaturated fatty acids generating a spectrum of bioactive lipid mediators. |
| Lonp1 | Medium | High | High | ATP-dependent serine protease that mediates the selective degradation of misfolded, unassembled or oxidatively damaged polypeptides. |
| Fga | Not detected | Not detected | Not detected | Major function in hemostasis as one of the primary components of blood clots. Functions during the early stages of wound repair to stabilize the lesion and guide cell migration during re-epithelialization. |
| Fgb | Not detected | Not detected | Not detected | Major function in hemostasis as one of the primary components of blood clots. Functions during the early stages of wound repair to stabilize the lesion and guide cell migration during re-epithelialization. |
| Fgg | Not detected | Not detected | Not detected | Together with fibrinogen alpha (FGA) and fibrinogen beta (FGB), polymerizes to form an insoluble fibrin matrix. Functions during the early stages of wound repair to stabilize the lesion and guide cell migration during re-epithelialization. |
| Apoh | Not detected | Not detected | Not detected | Binds to various kinds of negatively charged substances such as heparin, phospholipids, and dextran sulfate. Prevention of the intrinsic blood coagulation cascade by binding to phospholipids on the surface of damaged cells. |
| Crp | Not detected | Not detected | Not detected | A global transcription regulator. |
| Pf4 | Not detected | Not detected | Not detected | Released during platelet aggregation. Neutralizes the anticoagulant effect of heparin. Chemotactic for neutrophils and monocytes. |

Supplemental table 3. Blood coagulation and fibrinolytic system–related proteins unaffected by Muse cell injection: distribution and function.

Supplemental table 4. Quantitative proteomic dataset of lung tissue across experimental groups

(Excel file)

Supplemental Fig. 1: Stepwise extraction criteria and yield distribution of proteins in proteomic analysis. (a) A flowchart illustrating the stratified analysis of 2,694 proteins detected by LC-MS/MS. First, based on the comparison between the sham and hyperoxia-vehicle groups (p < 0.05, FDR < 0.10), proteins were classified into those significantly affected by hyperoxia (844 proteins) and those not significantly affected by hyperoxia (1,850 proteins). Each subset was then further divided based on whether the proteins were significantly affected by Muse cells (hyperoxia-vehicle vs. hyperoxia-Muse) or by non-Muse MSCs (hyperoxia -vehicle vs. hyperoxia-non-Muse). The resulting categories (I–XIII) cover all combinations of the “affected or unaffected by hyperoxia” and “affected or unaffected by Muse/non-Muse cells.” (b) A Venn diagram summarizing the classification of proteins in each subset. For example, among the 844 proteins affected by hyperoxia, 141 were significantly affected by Muse cell administration; of which, 56 were also significantly affected by the non-Muse cells. The 1,850 proteins not significantly affected by hyperoxia were classified in the same manner. (c) A summary table showing the number (and percentage) of proteins in each major classification, along with the calculated enrichment ratio.

Supplemental Fig. 2 (a) The number of ED1^+^ iNOS^+^ cell in the hyperoxia-induced lung injury model rats were elevated in the vehicle group compared with the sham group, and this increase was ameliorated by Muse cell administration. (n = 11 for sham, 12 for vehicle and 11 for Muse). *p < 0.05, ***p < 0.001. (b) Serum TNF-α levels in the hyperoxia rats were elevated in the vehicle group compared with the sham group, and this increase was ameliorated by Muse cell administration. (n = 7 for sham, 7 for vehicle and 8 for Muse). *p < 0.05, **p < 0.01

Supplemental Fig. 3: Relationships between blood-derived marker abundances and coagulation-related protein abundance in lung tissue proteomics. Scatter plots show (a) Hb_sum (HBB1 + HBB2 + HBA1/2) vs Coag_sum (FGA + FGB + FGG + F2), (b) EMI_sum (sum of erythrocyte marker proteins: HBB1, HBB2, HBA1/2, CA1, SLC4A1, GYPC, PRDX2) vs Coag_sum, and (c) PF4 abundance vs Coag_sum. (d) Spearman’s rank correlation coefficient (rs) and two‑sided p value a. Each dot represents an individual sample (Sham, Vehicle, non‑Muse, and Muse; n=8 per group). Protein abundances reported as not detected (n.d.; below the quantification limit) were imputed with the quantification limit value (55,671.01) prior to calculating summed indices.
